# Genome-Wide Identification and Comparative Analysis of *WOX* Genes in Four Euphorbiaceae Species and Their Expression Patterns in *Jatropha curcas*

**DOI:** 10.3389/fgene.2022.878554

**Published:** 2022-06-30

**Authors:** Zhanjun Wang, Qianwen Cai, Haimeng Xia, Bingqing Han, Minhui Li, Yue Wang, Minhui Zhu, Chunyan Jiao, Dandan Wang, Junjie Zhu, Wenya Yuan, Di Zhu, Congcong Xu, Hongyan Wang, Minghui Zhou, Xie Zhang, Jisen Shi, Jinhui Chen

**Affiliations:** ^1^ College of Life Sciences, Hefei Normal University, Hefei, China; ^2^ Key Laboratory of Forest Genetics and Biotechnology, Ministry of Education of China, Co-Innovation Center for the Sustainable Forestry in Southern China, Nanjing Forestry University, Nanjing, China; ^3^ State Key Laboratory of Utilization of Woody Oil Resource, Hunan Academy of Forestry, Changsha, China

**Keywords:** *WOX* genes, Euphorbiaceae, *Jatropha curcas*, bioinformatics analysis, gene expression

## Abstract

The WUSCHEL-related homeobox (WOX) proteins are widely distributed in plants and play important regulatory roles in growth and development processes such as embryonic development and organ development. Here, series of bioinformatics methods were utilized to unravel the structural basis and genetic hierarchy of *WOX* genes, followed by regulation of the *WOX* genes in four Euphorbiaceae species. A genome-wide survey identified 59 *WOX* genes in *Hevea brasiliensis* (*H. brasiliensis*: 20 genes), *Jatropha curcas* (*J. curcas*: 10 genes), *Manihot esculenta* (*M. esculenta*: 18 genes), and *Ricinus communis* (*R. communis*: 11 genes). The phylogenetic analysis revealed that these *WOX* members could be clustered into three close proximal clades, such as namely ancient, intermediate and modern/WUS clades. In addition, gene structures and conserved motif analyses further validated that the *WOX* genes were conserved within each phylogenetic clade. These results suggested the relationships among *WOX* members in the four Euphorbiaceae species. We found that *WOX* genes in *H. brasiliensis* and *M. esculenta* exhibit close genetic relationship with *J. curcas* and *R. communis*. Additionally, the presence of various *cis*-acting regulatory elements in the promoter of *J. curcas WOX* genes (*JcWOXs*) reflected distinct functions. These speculations were further validated with the differential expression profiles of various *JcWOXs* in seeds, reflecting the importance of two *JcWOX* genes (*JcWOX6* and *JcWOX13*) during plant growth and development. Our quantitative real-time PCR (qRT-PCR) analysis demonstrated that the *JcWOX11* gene plays an indispensable role in regulating plant callus. Taken together, the present study reports the comprehensive characteristics and relationships of *WOX* genes in four Euphorbiaceae species, providing new insights into their characterization.

## Introduction

The *WUSCHEL*-*related homeobox* (*WOX*) transcription factors are essential for cell fate determination, cell differentiation, regulation of various developmental processes and plant growth across the plant kingdom (Ueda et al., 2011; Costanzo et al., 2014; He et al., 2019). *WOX* genes are characterized by a conserved 60–66 residues long DNA-binding homeobox (HB) domain (helix 1-loop-helix 2-turn-helix 3) (Li et al., 2016). In addition, the tail region of the *WOX* family member are comprised of three specific conserved domains such as the WUS-box (TLXLFPXX), Ethylene-responsive element binding factor-associated Amphiphilic Repression (EAR) domain and acidic domain (Dolzblasz et al., 2016).

Haecker et al. (2004) categorized *WOX* genes of *Arabidopsis thaliana* (*A. thaliana*) into three major phylogenetic clades such as ancient clade (*WOX10*, *WOX13*, and *WOX14* subfamilies), intermediate clade (*WOX8*, *WOX9*, *WOX11*, and *WOX12* subfamilies) and modern/WUS clade (*WUS* and *WOX1-7* subfamilies) (Haecker et al., 2004). In the ancient clade, *WOX13* and *WOX14* play key roles in regulating flowers and fruits development and conducting tissues (Romera-Branchat et al., 2013; Costanzo et al., 2014). Genes *TaWOX8, TaWOX9* and *TaWOX12* from the intermediate clade could promote the immature callus proliferation in *Triticum aestivum* (*T. aestivum*) embryos (Shi et al., 2021). *WOX11*, regulated root *de novo* organogenesis in *A. thaliana* (Liu et al., 2014), showed high expression of gene ultimately increases nutrient uptake by callus in *Oryza Sativa* (*O. sativa*) (Wan Abdullah et al., 2021). In the modern/WUS clade, *WOX* genes are involved in the regulated development of various types of meristems and are differentially expressed in distinct species (Tvorogova et al., 2021). For instance, *WUS*, *WOX4*, and *WOX5* exhibit stem cell regulatory functions in the shoot apical meristem (SAM), vascular cambium (VCAM) and root apical meristem (RAM), respectively (Wang et al., 2011; Bueno et al., 2021). *CsWOX1* plays a major role in leaf vein morphology, leaf size and cell proliferation (Wang H. et al., 2020). Wu et al. (2007) reported that *WOX2* is necessary for the proper development of the embryonic apical region. *AtWOX2* positively regulates early embryonic development (Wu et al., 2007). In *Picea abies* (*P. abies*), *PaWOX2* and *PaWOX8/9* are expressed at high levels in the early growth stages of zygotic and somatic embryos (Palovaara et al., 2010). *PaWOX3* and *OsWOX3* could regulate the hormone expression levels in cells, hence, promoting cell division and development (Yoo et al., 2013; Yang et al., 2021). *TaWOX5* may be involved in root formation or development and hormone regulation during somatic embryogenesis in *T. aestivum* (Zhao et al., 2014). Overall, *WOX* genes play are not only responsible for the maintenance of stem cells in the apical meristem of shoots, roots, and the VCAM, the development of lateral organs, the formation of floral organs, the dynamic balance of embryonic development and postembryonic development, and the regulation of callus proliferation. To date, *WOX* gene members have been identified in various plant species, i.e., *Sorghum bicolor* (11 genes; Zhang et al., 2010), *Zea mays* (21 genes; Zhang et al., 2010), *Solanum lycopersicum* (10 genes; Dolzblasz et al., 2016), *Salix suchowensis* (15 genes; Wang et al., 2018), *Ricinus communis* (*R. communis*; http://castorbean.Jcvi. Org/index.php: 11 genes; Han et al., 2019), *Jatropha curcas* (*J. curcas*) (12 genes; Tang et al., 2019), and *Cucumis sativus* (11 genes; Han et al., 2021).

Euphorbiaceae, belongs to dicotyledonous angiosperms, which is widely distributed in tropical and subtropical regions (Webster, 1994). This family is comprised of approximately 300 genera and 800 species. The members of Euphorbiaceae including *M. esculenta* have extensive medicinal values, including antimicrobial, anti-inflammatory, anticancer and antioxidant activities (Webster, 1994; Santos-Silva et al., 2021). In addition, some Euphorbiaceae species have important economic value because they produce rubber, starch, and other compounds (Li et al., 2019). For example, *Hevea brasiliensis* (*H. brasiliensis*) is not only a perennial cross-pollinat tree with a long juvenile stage (Wang Y. et al., 2021) but also the main raw material for many industries, especially the tire industry (Supriya and Priyadarshan, 2019). Due to the high oil contents and adaptability to different environmental conditions, Euphorbiaceae plants such as *J. curcas* are considered as potential biodiesel sources in response to the current global energy crisis (Debnath and Bisen, 2008; Natarajan and Parani, 2011; Artimo et al., 2012; Maghuly and Laimer, 2013). As the main source of castor oil, *R. communis* has a high mineral oil accumulation capacity (Rehn et al., 2020). At present, the available whole-genome sequences of *H. brasiliensis* (Rahman et al., 2013), *J. curcas* (Hirakawa et al., 2012), *M. esculenta* (Prochnik et al., 2012) and *R. communis* (Chan et al., 2010) constitute an important foundation for current research and future molecular exploration. Although members of *WOX* genes have been identified and studied in many plant species, hitherto a comprehensive research on *WOX* genes of economically important Euphorbiaceae species is lacking.

In this study, the physicochemical properties, phylogenetic relationships, gene structure, conserved motifs, and codon usage bias of *WOX* genes in four Euphorbiaceae species were analyzed. Based on this analysis, overall molecular features of *WOX* genes were further clarified. Additionally, we studied *J. curcas* to identify the *cis*-acting elements in its *JcWOX* gene and determine its expression profile. The results provide further insights into the evolution and genetic relationships of four Euphorbiaceae species, as well as a basis for verifying the function of WOX transcription factors and screening *WOX* members in Euphorbiaceae species, which might be essential for plant growth and development.

## Materials and Methods

### Collection of Gene Sequences From Four Euphorbiaceae Species

The genome sequences of *J. curcas* were retrieved from the *Jatropha* Genome Database (http://www.kazusa.or.jp/jatropha/; JAT_r4.5; Hirakawa et al., 2012). The genome sequences of *H. brasiliensis* (Rahman et al., 2013), *M. esculenta* (Prochnik et al., 2012), and *R. communis* (Chan et al., 2010) were obtained from the National Center for Biotechnology Information (NCBI, https://www.ncbi.nlm.nih.gov/).

### Identification of *WOX* Genes in Four Euphorbiaceae Species

Redundant sequences were removed from the results obtained using the following two methods described below to identify *WOX* genes in four Euphorbiaceae species. (1) The hidden Markov model (HMM) file (PF00046) associated with WOX protein family-related domains was downloaded from the Pfam database (http://pfam.xfam.org/) (Cao et al., 2017). Using HMMER 3.0 software to analyze WOX proteins, the HMM file (PF00046) of four species of Euphorbiaceae was screened with an E-value cut off of 0.001. (2) 15 WOX protein sequences from *A. thaliana* were used as queries for alignment with the total protein sequences of the four Euphorbiaceae species by BLASTP with an E-value of 0.0001 to confirm the accuracy. The candidate sequences of *WOX* genes were detected using SMART (http://smart.embl-heidelberg.de/) to verify the presence of homeodomains. Ultimately, each *WOX* gene was assigned a unique name by BLASTP.

### Physicochemical Properties of WOX Proteins

The properties of WOX proteins, including their molecular weight (MW), isoelectric point (pI), instability index (II), aliphatic index (AI), were computed with ExPASy (https://web.expasy.org/protparam/; Artimo et al., 2012).

### Phylogenetic Classification and Gene Structure Analysis

All sequences from the four Euphorbiaceae species were subsequently aligned by DNAMAN software (version 6.0) to visualize the results. Moreover, multiple sequence alignment of all WOX protein sequences was performed by ClustalX 2.0 (Thompson et al., 1997). An interspecific phylogenetic tree containing the species was generated using the Neighbor-Joining (NJ) method with MEGA X software, including 1,000 bootstrap replicates (Kumar et al., 2018). In addition, the exon and intron structures of all *WOX* genes were obtained from the online Gene Structure Display Server (GSDS; http://gsds.cbi.pku.edu.cn; Hu et al., 2015).

### Conserved Motif Analysis

The conserved motifs of WOX proteins from the four Euphorbiaceae species were analyzed by MEME online server (http://meme-suite.org/). The maximum number of identified motifs was set to 25 (E-value = 0.0001; Li et al., 2018).

### Codon Usage Bias Analysis

The codon usage bias of *WOX* genes in the four Euphorbiaceae species was analyzed using CodonW software (version 1.4.2). The relative synonymous codon usage (RSCU) of *WOX* genes in these species was calculated in such a way that RSCU value >1 represents a codon with positive bias; an RSCU value = 1 indicates no bias (Wang Z. et al., 2021). The relative frequencies of synonymous codons (RFSCs) in the *J. curcas WOX* genes (*JcWOXs*) were also calculated. When the RFSC value exceeds 60% or is 0.50 times higher than the average frequency of synonymous codons, the codon is a high-frequency codon (Zhou et al., 2007a). Furthermore, a comparative analysis of the codon usage frequency among *WOX* genes of the four Euphorbiaceae species and four representative species, *A. thaliana*, *Nicotiana tabacum* (*N. tabacum*), *Populus trichocarpa* (*P. trichocarpa*), and *O. sativa*, was performed. The ratio ranged from 0.50 to 2.00 reflects that the codon usage bias of *WOX* genes is highly similar to that of the representative plant species (Zhou et al., 2007b).

### *Cis*-Acting Elements of *JcWOXs*

The genomic sequences located 2000 bp upstream from the initiation codon (ATG) were considered as the promoter fragments. The promoter sequences of *JcWOXs* were downloaded from the *Jatropha* Genome Database (Hirakawa et al., 2012). Meanwhile, PlantCARE online sever (http://bioinformatics.psb.ugent.be/webtools/plantcare/html/) was used to analyze potential *cis*-acting elements (Lescot et al., 2002).

### Spatial Expression Profiles of *JcWOXs*

*J. curcas* RNA transcriptome data from three different tissues (leaves, roots, and seeds) were downloaded from the Sequence Read Archive (SRA) database to investigate the spatial expression characteristics of *JcWOXs* (Wu et al., 2015; Zou et al., 2016). The spatial expression profiles of each identified *JcWOX* in three different tissues were determined using three transcriptome datasets. The spatial characteristics of 10 *JcWOXs* were explored using the R library heatmap, and the trends were ultimately presented as color changes. Detailed information of the three transcriptome datasets was provided in Supplementary Table S1.

### Temporal Expression Profiles of *JcWOXs*

The RNA transcriptome data collected at seven developmental stages (14, 19, 25, 29, 35, 41, and 45 days after pollination-DAP) of seeds were downloaded following the spatial expression profiles of *JcWOXs* (Jiang et al., 2012). The temporal characteristics of the 10 *JcWOXs* were explored by generating the heatmap. The detailed data information of the seven transcriptomes is presented in Supplementary Table S1.

### Expression Profiles of *JcWOXs* in Calli

*J. curcas* calli were selected as experimental materials to test whether *WOX* gene has functional conservation in regulating callus proliferation. The third leaf from the top of Guangxi *J. curcas* was selected as the explant. Next, the explants were disinfected by washing with water for 0.50–1.00 min, followed by soaking in 70% ethanol for 15 s and 3% NaClO solution for 12–16 min. Callus induction was performed by inoculating the explant in dorsal contact medium supplemented with MS, BAP (+0.80 mg/L), TDZ (+0.60 mg/L) and NAA (+0.10 mg/L). Explants were cultured in the dark at 25°C. The whole cycle of callus culture was approximately 42 days, which was divided into three stages: 14 days (S1), 28 days (S2), and 42 days (S3), as illustrated in Supplementary Figure S1. With obvious differences in the growth states and callus characteristics were selected as samples. Three biological replicates of each sample were analyzed. All samples were collected and cryo-preserved in liquid nitrogen.

Total RNA was extracted from *J. curcas* callus was extracted using the FastPure plant total RNA isolation kit (RC401). The RNA concentration was determined using a Nanodrop-2000 spectrophotometer (Thermo, Inc.). The integrity of total RNA was detected by performing gel electrophoresis. First-strand cDNAs were synthesized using the reverse transcriptase method with the HiScriptR III 1st Strand cDNA Synthesis Kit (+ gDNA wiper) (R312-01/02). Additionally, the samples in triplicate were analyzed with qRT-PCR utilizing AceQ qPCR SYBR Green Master Mix (without ROX), as previously described by Wang D. et al. (2020). Four reference genes (*JcGAPDH*, *JcEF1α*, *JcActin*, and *JcTUB8*) were selected as candidate reference genes according to Zhang et al. (2013), and *JcGAPDH* and *JcActin* were determined as reference genes by using semi-quantitative RT-PCR (Liu et al., 2010). Based on the coding DNA sequences (CDSs) of *WOX* gene from *J. curcas*, primers were designed using Oligo7 and SnapGene (Supplementary Table S2).

## Results

### Identification of *WOX* Genes in Four Euphorbiaceae Species

Both HMMER and local BLAST searches were performed simultaneously with the HMM file (PF00046), and the sequences of 15 members of the *WOX* family in *A. thaliana* were used as templates to identify all possible WOX proteins in the four Euphorbiaceae species and confirm the accuracy of the identification. Ultimately, 59 *WOX* genes were identified in the genomes of the four Euphorbiaceae species such as, 20 in *H. brasiliensis*, 10 in *J. curcas*, 18 in *M. esculenta*, and 11 in *R. communis*. These *WOX* genes were named by BLASTP (Supplementary Table S3).

### Analysis of Physicochemical Properties

Next, the candidate *WOX* gene sequences were isolated, followed by analyzing the physicochemical properties of their corresponding proteins utilizing by ExPASy. As shown in Supplementary Table S3, their protein lengths and predicted MW varied little, and few differences in their pI were observed. For *H. brasiliensis*, the WOX proteins were 185–398 residues long, corresponding MW ranged from 21,346.99 to 44,058.10 Da, and their pI values ranged from 5.63 (HbWOX11b) to 9.51 (HbWOX4b). For *J. curcas*, the corresponding WOX proteins ranged in length from 190 (JcWOX7) to 392 (JcWOX1) aa, their MW ranged from 21,747.21 to 43,900.70 Da, and their pI values ranged from 5.15 (JcWOX14) to 9.51 (JcWOX4). WOX proteins in *M. esculenta* were 182 to 391 residues long, with the pI values ranging from 5.61 (MeWUSb) to 9.40 (MeWOX4a). For *R. communis*, the WOX proteins ranged in length from 192 to 401 residues long, and their pI values ranged from 5.35 (RcWOX14) to 9.42 (RcWOX1a). Therefore, the physicochemical properties of the WOX proteins in the four Euphorbiaceae species were similar. The hydropathicity index represents the hydrophilicity of a protein (Li et al., 2016). The AI ranged from 44.34 to 82.58, indicating that WOX proteins are thermally stable. As shown in Supplementary Table S3, the hydrophilicity index of 59 members of the WOX protein families was negative, suggesting that the members of the four WOX protein families of the Euphorbiaceae species were hydrophilic.

### Phylogenetic Classification and Gene Structure Analysis

According to the visualized of sequence alignment (Supplementary Figure S2), the homeodomain of WOX protein of *A. thaliana* and the four Euphorbiaceae species presented high conserved structures. The number of amino acid residues in WOX homeodomain fluctuated slightly, from 63 to 66. These results proved that the WOX proteins of four Euphorbiaceae species were highly conserved, further indicating that the identified *WOX* genes were correctly identified.

**FIGURE 1:**
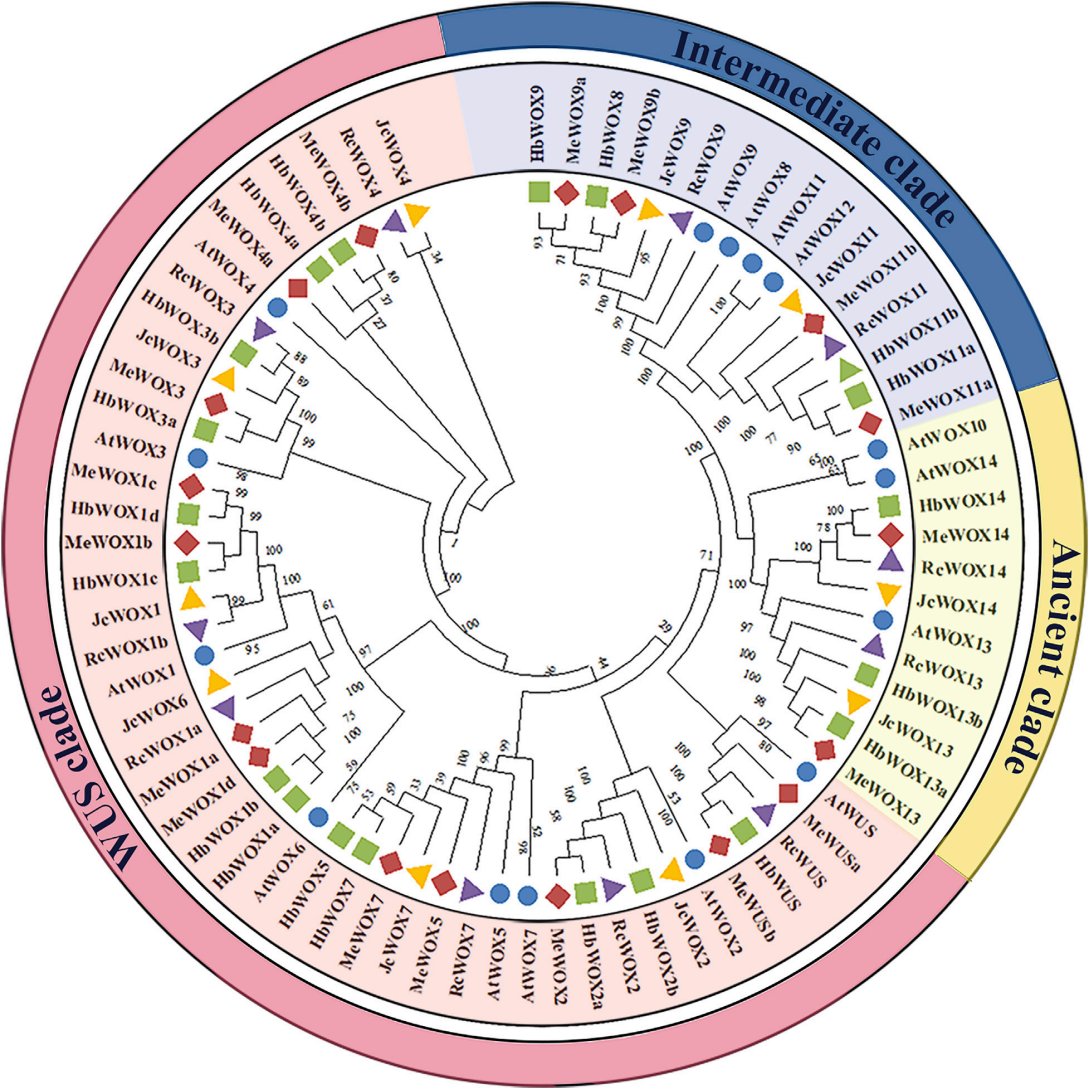
Phylogenetic tree of *WOX* genes from four Euphorbiaceae species and *Arabidopsis thaliana*. The five plant species that are members of the *WOX* gene family can be divided into three main clade classes, the ancient clade includes three subfamilies, the intermediate clade includes three subfamilies, and the modern/WUS clade contains eight subfamilies.

In order to compare the evolutionary relationships of *WOX* genes of *A. thaliana* and the four Euphorbiaceae species, NJ phylogenetic trees of 74 WOX proteins from *A. thaliana* (15), *H. brasiliensis* (20), *J. curcas* (10), *M. esculenta* (18), and *R. communis* (11) were constructed with MEGA X software to compare the evolutionary relationships of *WOX* genes from *A. thaliana* and the four Euphorbiaceae species. It was observed from Figure 1 that *WOX* genes from the five species were classified into three major clades, which is consistent with the previously known clades. For instance, ancient clade (WOX10, WOX13, and WOX14 subfamilies), intermediate clade (WOX8, WOX9, and WOX11), and modern/WUS clade contains eight subfamilies (WUS and WOX1-7). The number of *WOX* genes in the modern/WUS clade (38 genes, 64.4%) was greater than that in the intermediate (12 genes, 20.3%) and the ancient clades (nine genes, 15.3%). Moreover, phylogenetic tree analysis reflected that the homologous genes lies in close proximity, suggesting that the *WOX* genes in Euphorbiaceae species were highly evolutionarily conserved. Furthermore, members of the WOX transcription factors in *H. brasiliensis* and *M. esculenta* were located close to each other in the evolutionary tree.

**FIGURE 2:**
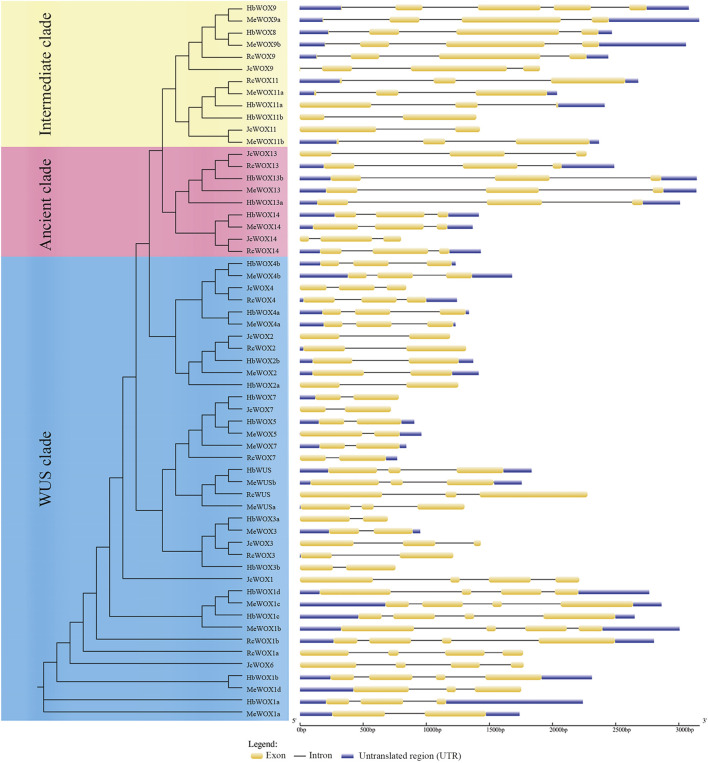
*WOX* gene structure according to the phylogenetic relationship. The composition of the *WOX* gene includes exons, introns, and untranslated regions (UTRs).

Structure analysis of 59 *WOX* genes was performed with GSDS. *WOX* gene is composed of exons, introns, and untranslated regions (UTRs), as illustrated in Figure 2. The number of exons in each WOX gene ranged from two to four. The *WOX* genes members in the same subclade usually presented similar exon–intron patterns. For example, the members of the closely related modern clade i.e., *HbWOX7*, *JcWOX7*, *HbWOX5*, *MeWOX5*, *MeWOX7*, and *RcWOX7* exhibit similar gene lengths with two exons. In addition, members of the ancient clade had two intron insertion sites, while members of the intermediate and modern/WUS clades exhibited different intron insertion patterns, and the number of introns ranged from one to four. Moreover, five genes (*HbWOX9*, *MeWOX9a*, *MeWOX9b*, *HbWOX13b*, and *MeWOX13*) were more than 3 kb. Interestingly, most *JcWOX* and *RcWOX* members were located in adjacent regions and had similar gene structures, showing that the similarity between *J. curcas* and *R. communis* species is high and the kinship is close. The same phenomenon was also found in most of the *HbWOX* and *MeWOX* members. Collectively, these results indicated that similar gene structures also reflect the conservation of *WOX* gene members and their evolutionary relationships.

### Conserved Motif Analyses

**FIGURE 3:**
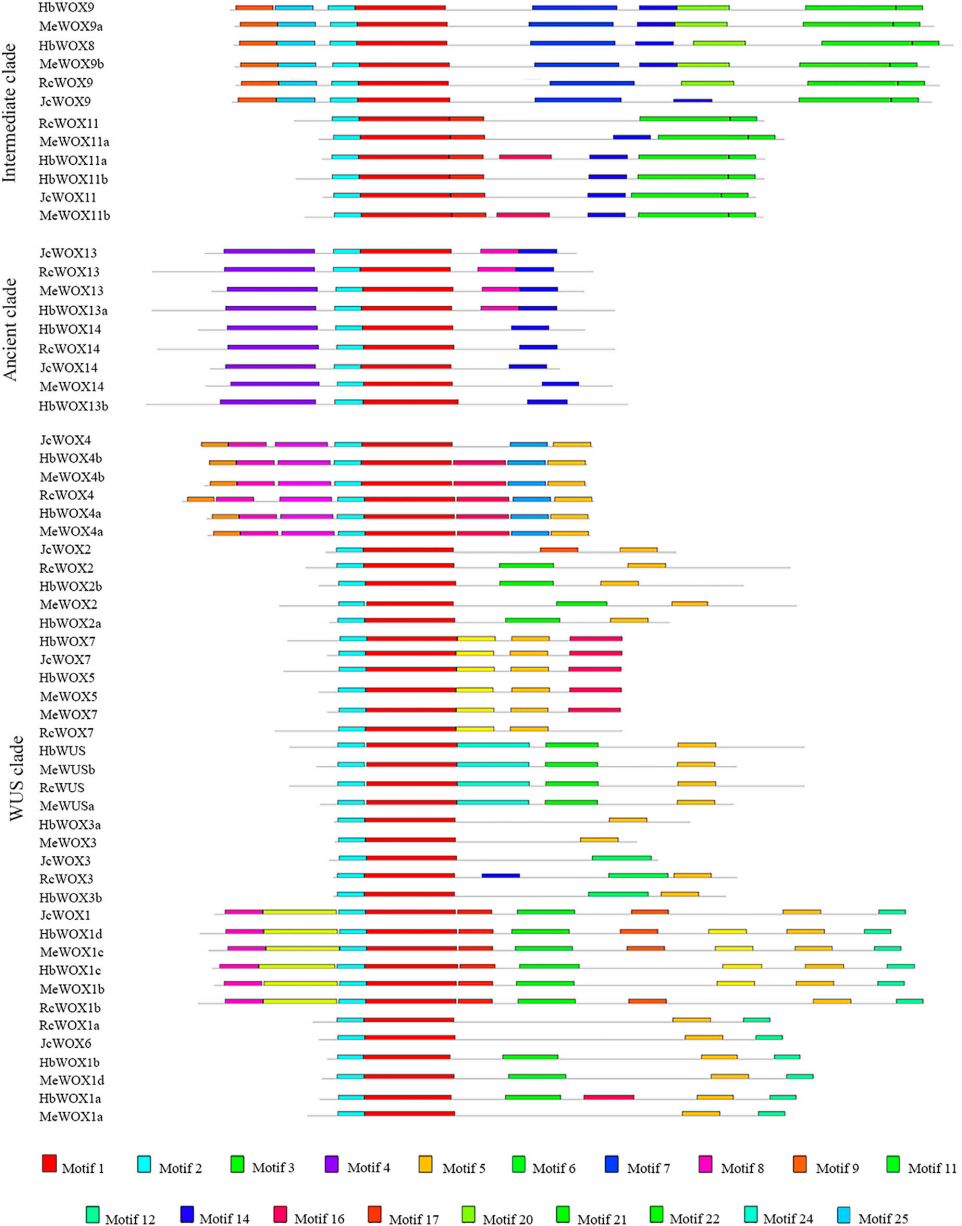
Distribution of conserved motifs identified among four Euphorbiaceae species. 25 conserved motifs identified in 59 WOX proteins, which can also be divided into ancient, intermediate, and modern/WUS clades according to the phylogenetic tree.

The WOX family typically contains additional conserved motifs that likely have involved in different functions. 25 conserved motifs were identified among the 59 WOX proteins in the four Euphorbiaceae species using the MEME online portal to obtain a comprehensive understanding of the structural features and relationships of the WOX proteins. The phylogenetic tree (Figure 3) was divided into ancient, intermediate, and modern/WUS clades. Among the 25 putative motifs, motif 1 and motif 2 were present in all WOX protein sequences, indicating that motifs 1 and 2 are characteristic domains of the proteins encoded by 59 *WOX* genes. Structural analysis demonstrated that proteins corresponding HbWOX3a and MeWOX3 exhibit only two motifs while HbWOX1d and MeWOX1c possess 10 motifs. With the exception of JcWOX3, motif 5 was present at a high frequency in nearly all WOX proteins in the modern/WUS clade, but this motif was not present in the intermediate clade or ancient clade, consistent with the distribution of the WUS box (TLXLFPXX). In particular, motif 4 existed only in the ancient clade. Furthermore, the conserved motifs of *JcWOX* and *RcWOX* were highly similar, as observed for *HbWOX* and *MeWOX*. The *WOX* members within the same clade had similar motif structures, suggesting that the homologous *WOX* genes in different plant species are closely related. These results are consistent with previously reported phylogenetic tree analysis and could further strengthens the classification of WOX subfamily members.

### Codon Usage Bias Analysis

CodonW software was used to study the codon usage bias in the 59 *WOX* genes from the four Euphorbiaceae species. As shown in Table 1, the codon usage of *WOX* genes across the Euphorbiaceae species was quite conserved. According to the RSCU values, strong commonalities were detected in *WOX* genes from the four Euphorbiaceae species. 19, 23, and two identical codons displayed positive bias, negative bias and no bias, respectively; the remaining 20 codons differed in bias. The strongest positive bias in all species presents same codons (AGA), however, the codons with the strongest negative bias were different such as *J. curcas* (CGC) and *M. esculenta* (CGC), *H. brasiliensis* (GCG) and *R. communis* (ACG). An analysis of the RFSCs of the *JcWOXs* revealed four identical high-frequency codons from *WOX* genes of the four Euphorbiaceae species: GCA, GAU, CCA, and AGA. However, some differences in the high-frequency codons were observed. For instance, CUU, CAA, and ACU were high-frequency codons in *M. esculenta* but not in the other three species. Additionally, the codon usage frequency ratios between the four Euphorbiaceae species and four model organisms were analyzed (Table 2). The ratio of six codons (GCU, CGC, UCG, UAA, UAG, and UGA; GCU, GAU, CGC, UAA, UAG, and UGA) were greater than 2.00 and lower than 0.50 in the *JcWOX* genes than in the *A. thaliana* and *P. trichocarpa* genes, respectively. Comparative analysis reflected, five similar codons (GCC, CGG, UAA, UAG, and UGA) in *RcWOX* genes and *P. trichocarpa* genes, five codons (GCC, CGC, UAA, UAG, and UGA) showed differences in the comparison between the *MeWOX* genes and *N. tabacum* genes, and six codons (GCG, UUG, CGG, UAA, UAG, and UGA) showed differences in the comparison between the *HbWOX* genes and *P. trichocarpa* genes. Hence, we considered *P. trichocarpa* to be the optimal choice for comparing exogenous *WOX* genes in *J. curcas*, *R. communis*, and *H. brasiliensis* and *N. tabacum* the optimal choice for comparing exogenous *WOX* genes in *M. esculenta*.

**TABLE 1:** RSCU and RFSC of codons in *WOX* genes of four Euphorbiaceae species.

Amino acid	Codon	*HbWOX*		*JcWOX*		*MeWOX*		*RcWOX*	
		RSCU	RFSC	RSCU	RFSC	RSCU	RFSC	RSCU	RFSC
A (Ala)	GCU	**1.27**	31.66	0.90	22.52	**1.37**	34.26	**1.33**	33.33
	GCC	0.76	18.92	0.54	13.51	0.51	12.75	0.43	10.64
	GCA	**1.84**	***45.95***	**2.09**	***52.26***	**1.83**	***45.82***	**1.90**	***47.52***
	GCG	0.14	3.47	0.47	11.71	0.29	7.17	0.34	8.51
C (Cys)	UGU	**1.03**	51.55	**1.13**	56.60	0.86	43.09	0.88	43.94
	UGC	0.97	48.45	0.87	43.40	**1.14**	56.91	**1.12**	56.06
D (Asp)	GAU	**1.45**	***72.49***	**1.28**	***63.86***	**1.37**	***63.33***	**1.45**	***72.45***
	GAC	0.55	27.51	0.72	36.14	0.51	36.67	0.55	27.55
E (Glu)	GAA	**1.20**	59.89	**1.35**	***67.35***	**1.83**	***62.87***	**1.27**	***63.64***
	GAG	0.80	40.11	0.65	32.65	0.29	37.13	0.73	36.36
F (Phe)	UUU	1.00	50.22	0.98	49.17	**1.05**	52.27	**1.02**	51.16
	UUC	1.00	49.78	**1.02**	50.83	0.95	47.73	0.98	48.84
G (Gly)	GGU	**1.17**	29.28	**1.35**	33.87	**1.06**	26.53	**1.16**	29.03
	GGC	0.78	19.42	0.52	12.90	0.72	17.96	0.77	19.35
	GGA	**1.37**	34.20	**1.52**	***37.91***	**1.36**	33.88	**1.34**	33.56
	GGG	0.68	17.10	0.61	15.32	0.87	21.63	0.72	18.06
H (His)	CAU	0.95	47.37	**1.02**	50.79	**1.05**	***64.43***	**1.27**	***63.53***
	CAC	**1.05**	52.63	0.98	49.21	0.90	35.57	0.73	36.47
I (Ile)	AUU	**1.11**	37.15	**1.10**	36.62	**1.09**	36.44	**1.11**	37.02
	AUC	**1.08**	35.97	0.93	30.99	**1.09**	36.44	**1.07**	35.71
	AUA	0.81	26.88	0.97	32.39	0.81	27.12	0.82	27.27
K (Lys)	AAA	0.84	41.83	0.96	47.95	**1.57**	46.34	0.94	47.19
	AAG	**1.16**	58.17	**1.04**	52.05	0.43	53.66	**1.06**	52.81
L (Leu)	UUA	0.73	12.14	0.92	15.35	0.58	9.64	0.74	12.26
	UUG	0.87	14.47	0.85	14.17	0.92	15.30	**1.06**	17.62
	CUU	**1.69**	***28.18***	**1.84**	***30.72***	**1.47**	24.53	**1.52**	***25.29***
	CUC	**1.02**	17.05	0.83	13.78	**1.17**	19.50	0.78	13.03
	CUA	0.73	12.14	0.59	9.84	0.72	11.95	0.90	14.94
	CUG	0.96	16.02	0.97	16.14	**1.14**	19.08	**1.01**	16.86
M (Met)	AUG	1.00	100.00	1.00	100.00	1.00	100.00	1.00	100.00
N (Asn)	AAU	**1.19**	59.32	**1.16**	58.00	**1.18**	50.19	0.95	47.59
	AAC	0.81	40.68	0.84	42.00	0.82	49.81	**1.05**	52.41
P (Pro)	CCU	**1.39**	34.80	**1.12**	27.91	**1.05**	26.25	**1.33**	33.33
	CCC	0.53	13.17	0.68	17.05	0.90	22.50	0.68	17.01
	CCA	**1.81**	***45.13***	**1.71**	***42.64***	**1.75**	***43.75***	**1.74**	***43.54***
	CCG	0.28	6.90	0.50	12.40	0.30	7.50	0.24	6.12
Q (Gln)	CAA	**1.21**	***60.68***	**1.25**	***62.58***	**1.75**	58.77	**1.24**	***61.88***
	CAG	0.79	39.32	0.75	37.42	0.30	41.23	0.76	38.12
R (Arg)	CGU	0.61	10.14	0.53	8.82	0.59	9.82	0.42	7.06
	CGC	0.37	6.20	0.18	2.94	0.18	2.98	0.32	5.29
	CGA	0.49	8.17	0.42	7.06	0.68	11.31	0.32	5.29
	CGG	0.25	4.23	0.46	7.65	0.27	4.46	0.25	4.12
	AGA	**2.74**	***45.63***	**3.25**	***54.12***	**2.68**	***44.64***	**3.28**	***54.71***
	AGG	**1.54**	***25.63***	**1.16**	19.41	**1.61**	***26.79***	**1.41**	23.53
S (Ser)	UCU	**1.44**	23.94	**1.40**	23.31	**1.29**	21.49	**1.31**	21.77
	UCC	0.72	11.97	0.94	15.68	0.88	14.68	0.83	13.88
	UCA	**1.43**	23.77	**1.37**	22.88	**1.54**	***25.74***	**1.61**	***26.81***
	UCG	0.25	4.23	0.25	4.24	0.27	4.47	0.25	4.10
	AGU	0.98	16.37	0.71	11.86	0.88	14.68	0.93	15.46
	AGC	**1.18**	19.72	**1.32**	22.03	**1.14**	18.94	**1.08**	17.98
T (Thr)	ACU	**1.50**	***37.50***	**1.51**	***37.80***	**1.18**	29.57	**1.62**	***40.47***
	ACC	0.80	20.12	0.78	19.51	0.82	20.60	0.71	17.67
	ACA	**1.35**	33.84	**1.39**	34.76	**1.57**	***39.20***	**1.47**	36.74
	ACG	0.34	8.54	0.32	7.93	0.43	10.63	0.20	5.12
V (Val)	GUU	**1.47**	36.81	**1.46**	36.43	**1.37**	34.34	**1.24**	31.01
	GUC	0.60	15.00	0.85	21.19	0.57	14.35	0.68	17.05
	GUA	0.75	18.64	0.58	14.41	0.71	17.83	**1.15**	28.68
	GUG	**1.18**	29.55	**1.12**	27.97	**1.34**	33.48	0.93	23.26
W (Trp)	UGG	1.00	100.00	1.00	100.00	1.00	100.00	1.00	100.00
Y (Tyr)	UAU	**1.07**	53.38	0.98	49.18	**1.29**	49.14	**1.10**	55.07
	UAC	0.93	46.62	**1.02**	50.82	0.88	50.86	0.90	44.93
TER	UGA	**1.95**	***65.00***	**1.38**	2.54	**1.55**	***51.79***	**1.58**	***52.81***
	UAA	0.79	26.25	0.95	***57.00***	**1.54**	29.50	0.91	30.34
	UAG	0.26	8.75	0.67	40.46	0.27	18.71	0.51	16.85

Bold in the table means RSCU >1; the bold and italic numbers in the table indicate the RFSC value of high frequency codon.

**TABLE 2:** Comparison of codon usage frequency of *WOX* genes of four Euphorbiaceae species and four representative plant genomes.

Amino acid	Codon	*HbWOX/At*	*HbWOX/Nt*	*HbWOX/Pt*	*HbWOX/Os*	*JcWOX/At*	*JcWOX/Nt*	*JcWOX/Pt*	*JcWOX/Os*	*MeWOX/At*	*MeWOX/Nt*	*MeWOX/Pt*	*MeWOX/Os*	*RcWOX/At*	*RcWOX/Nt*	*RcWOX/Pt*	*RcWOX/Os*
A (Ala)	GCU	0.53	*0.48*	0.68	0.76	*0.33*	*0.30*	*0.43*	*0.48*	0.60	0.54	0.77	0.86	0.54	*0.49*	0.70	0.78
	GCC	0.87	0.72	0.91	*0.29*	0.55	*0.45*	0.58	*0.18*	0.61	*0.50*	0.64	*0.20*	*0.48*	*0.39*	*0.50*	*0.16*
	GCA	1.24	0.94	1.08	1.26	1.25	0.94	1.08	1.26	1.29	0.98	1.12	1.31	1.25	0.95	1.09	1.27
	GCG	*0.18*	*0.28*	*0.44*	*0.06*	0.54	0.84	1.32	*0.18*	*0.39*	0.61	0.96	*0.13*	*0.44*	0.68	1.06	*0.15*
C (Cys)	UGU	0.87	0.93	0.82	1.47	1.07	1.15	1.01	1.82	0.99	1.06	0.93	1.68	0.90	0.97	0.85	1.53
	UGC	1.19	1.19	0.97	0.69	1.20	1.20	0.97	0.70	1.91	1.91	1.55	1.11	1.68	1.68	1.36	0.98
D (Asp)	GAU	0.68	0.68	0.60	0.99	0.54	0.54	*0.48*	0.79	0.51	0.51	*0.45*	0.74	0.63	0.63	0.56	0.92
	GAC	0.55	0.56	0.66	*0.34*	0.66	0.67	0.79	*0.40*	0.63	0.64	0.76	*0.38*	0.51	0.52	0.62	*0.31*
E (Glu)	GAA	1.16	1.11	0.98	1.85	1.09	1.03	0.92	1.72	1.11	1.05	0.94	1.76	1.07	1.02	0.91	1.70
	GAG	0.83	0.91	0.82	0.69	0.56	0.61	0.56	*0.47*	0.70	0.76	0.69	0.58	0.65	0.71	0.64	0.54
F (Phe)	UUU	0.95	0.82	0.80	1.58	1.02	0.88	0.86	1.69	1.04	0.90	0.87	1.73	0.99	0.86	0.83	1.65
	UUC	0.99	1.14	1.17	0.91	1.11	1.27	1.31	1.02	1.00	1.15	1.18	0.92	1.00	1.15	1.18	0.92
G (Gly)	GGU	0.83	0.83	1.03	1.25	0.71	0.71	0.88	1.07	0.58	0.57	0.71	0.86	0.66	0.66	0.82	1.00
	GGC	1.33	1.09	1.20	*0.42*	0.65	0.54	0.59	*0.20*	0.94	0.77	0.85	*0.29*	1.07	0.88	0.96	*0.33*
	GGA	0.89	0.93	0.95	1.36	0.73	0.76	0.78	1.11	0.67	0.70	0.72	1.03	0.70	0.73	0.75	1.07
	GGG	1.06	1.03	0.94	0.63	0.70	0.68	0.62	*0.42*	1.02	0.99	0.91	0.61	0.90	0.87	0.80	0.54
H (His)	CAU	1.07	1.11	0.93	1.31	0.87	0.90	0.76	1.06	1.37	1.41	1.19	1.67	1.28	1.32	1.11	1.56
	CAC	1.89	1.89	1.98	1.19	1.34	1.34	1.40	0.84	1.20	1.20	1.26	0.75	1.17	1.17	1.22	0.74
I (Ile)	AUU	0.80	0.62	0.59	1.21	0.91	0.70	0.67	1.38	0.82	0.64	0.60	1.25	0.87	0.67	0.64	1.31
	AUC	0.90	1.20	1.09	0.86	0.89	1.19	1.09	0.85	0.96	1.27	1.16	0.91	0.97	1.30	1.18	0.93
	AUA	0.99	0.89	0.83	1.41	1.37	1.24	1.15	1.97	1.05	0.94	0.88	1.50	1.09	0.98	0.92	1.56
K (Lys)	AAA	0.65	0.62	0.59	1.26	1.00	0.95	0.90	1.93	0.85	0.80	0.77	1.63	0.89	0.84	0.81	1.72
	AAG	0.86	0.84	0.86	0.87	1.02	1.00	1.03	1.04	0.93	0.90	0.93	0.94	0.94	0.92	0.95	0.95
L (Leu)	UUA	0.68	0.64	0.58	1.41	1.15	1.09	0.98	*2.40*	0.71	0.67	0.61	1.48	0.82	0.78	0.70	1.72
	UUG	*0.49*	*0.46*	*0.40*	0.70	0.65	0.61	0.53	0.92	0.69	0.64	0.56	0.98	0.72	0.68	0.59	1.02
	CUU	0.83	0.83	0.69	1.31	1.22	1.22	1.01	1.93	0.95	0.96	0.79	1.51	0.90	0.90	0.74	1.42
	CUC	0.75	0.98	0.86	*0.47*	0.82	1.07	0.93	0.51	1.14	1.49	1.30	0.71	0.69	0.90	0.79	*0.43*
	CUA	0.87	0.91	0.71	1.12	0.95	1.00	0.78	1.22	1.13	1.19	0.93	1.45	1.29	1.36	1.06	1.66
	CUG	1.16	1.11	0.77	0.54	1.57	1.51	1.05	0.73	1.83	1.75	1.22	0.85	1.47	1.41	0.98	0.69
M (Met)	AUG	1.00	0.98	1.04	1.03	0.84	0.83	0.88	0.87	1.12	1.09	1.16	1.15	0.99	0.97	1.03	1.02
N (Asn)	AAU	1.57	1.25	1.26	*2.31*	1.47	1.17	1.18	*2.17*	1.15	0.91	0.92	1.69	1.16	0.92	0.93	1.71
	AAC	1.15	1.34	1.54	1.29	1.13	1.32	1.52	1.28	1.21	1.42	1.63	1.37	1.36	1.59	1.83	1.54
P (Pro)	CCU	1.09	1.09	1.27	1.49	0.72	0.72	0.85	1.00	0.66	0.66	0.77	0.91	0.86	0.86	1.00	1.18
	CCC	1.45	1.16	1.48	0.63	1.56	1.25	1.59	0.68	*2.00*	1.61	*2.04*	0.88	1.54	1.24	1.57	0.68
	CCA	1.64	1.33	1.58	1.85	1.28	1.04	1.24	1.46	1.28	1.04	1.24	1.45	1.30	1.06	1.25	1.48
	CCG	*0.47*	0.80	1.01	*0.22*	0.70	1.20	1.50	*0.33*	*0.41*	0.71	0.88	*0.20*	*0.34*	0.59	0.74	*0.16*
Q (Gln)	CAA	*2.01*	1.88	1.86	*2.88*	1.88	1.76	1.74	*2.70*	1.83	1.72	1.70	*2.64*	1.89	1.77	1.75	*2.72*
	CAG	1.66	1.68	1.43	1.21	1.43	1.45	1.24	1.05	1.64	1.66	1.42	1.20	1.49	1.51	1.28	1.09
R (Arg)	CGU	0.73	0.88	0.89	0.91	0.63	0.75	0.76	0.78	0.72	0.86	0.88	0.90	*0.44*	0.52	0.53	0.55
	CGC	1.06	1.03	0.89	*0.25*	*0.49*	*0.48*	*0.42*	*0.12*	0.52	*0.50*	*0.44*	*0.12*	0.78	0.76	0.65	*0.18*
	CGA	0.84	1.00	0.96	0.83	0.72	0.85	0.82	0.70	1.19	1.41	1.36	1.17	*0.47*	0.56	0.54	*0.46*
	CGG	0.56	0.74	*0.48*	*0.20*	1.00	1.32	0.86	*0.36*	0.60	0.80	0.52	*0.22*	*0.47*	0.62	*0.40*	*0.17*
	AGA	1.56	1.85	1.51	*2.82*	1.82	*2.16*	1.76	*3.29*	1.55	1.84	1.50	*2.81*	1.60	1.90	1.55	*2.90*
	AGG	1.51	1.36	1.31	1.04	1.13	1.02	0.98	0.78	1.61	1.45	1.39	1.11	1.19	1.07	1.03	0.82
S (Ser)	AGU	1.21	1.28	1.13	1.93	0.75	0.79	0.70	1.20	0.97	1.02	0.90	1.54	1.15	1.21	1.06	1.82
	AGC	1.81	*2.05*	1.81	1.28	1.73	1.95	1.73	1.22	1.55	1.75	1.55	1.09	1.65	1.87	1.65	1.17
	UCU	0.99	1.24	1.21	1.96	0.82	1.03	1.01	1.63	0.79	0.99	0.97	1.56	0.90	1.13	1.10	1.78
	UCC	1.11	1.22	1.45	0.76	1.24	1.36	1.62	0.85	1.21	1.33	1.58	0.83	1.29	1.41	1.67	0.88
	UCA	1.35	1.40	1.25	1.99	1.11	1.15	1.03	1.64	1.30	1.35	1.21	1.92	1.52	1.58	1.41	*2.24*
	UCG	*0.47*	0.83	0.88	*0.36*	*0.40*	0.71	0.75	*0.31*	*0.44*	0.78	0.83	*0.34*	*0.46*	0.80	0.85	*0.35*
T (Thr)	ACU	1.29	1.11	1.56	*2.12*	1.33	1.15	1.62	*2.20*	1.00	0.86	1.21	1.65	1.63	1.40	1.98	*2.69*
	ACC	1.17	1.24	1.45	0.81	1.17	1.24	1.45	0.81	1.18	1.26	1.47	0.82	1.21	1.28	1.50	0.83
	ACA	1.29	1.17	1.33	1.75	1.36	1.23	1.40	1.85	1.48	1.33	1.52	*2.00*	1.65	1.49	1.69	*2.23*
	ACG	0.66	1.14	1.16	*0.45*	0.63	1.09	1.11	*0.43*	0.82	1.40	1.43	0.55	*0.47*	0.80	0.82	*0.32*
V (Val)	GUU	0.54	0.55	0.61	0.96	0.59	0.60	0.67	1.04	0.57	0.58	0.64	1.00	*0.48*	*0.49*	0.54	0.84
	GUC	*0.47*	0.54	0.53	*0.30*	0.73	0.85	0.83	*0.47*	0.51	0.58	0.57	*0.32*	0.56	0.65	0.64	*0.36*
	GUA	0.76	0.66	0.73	1.10	0.65	0.56	0.63	0.94	0.81	0.71	0.79	1.19	1.22	1.06	1.19	1.78
	GUG	0.68	0.71	0.70	*0.49*	0.71	0.74	0.73	0.51	0.87	0.91	0.90	0.62	0.56	0.59	0.58	*0.40*
W (Trp)	UGG	1.33	1.36	1.20	1.21	1.68	1.73	1.51	1.53	1.48	1.51	1.33	1.34	1.44	1.48	1.30	1.30
Y (Tyr)	UAU	0.99	0.81	0.89	1.44	0.77	0.63	0.69	1.13	0.77	0.63	0.69	1.12	0.85	0.70	0.76	1.24
	UAC	0.92	0.93	1.33	0.84	0.85	0.86	1.23	0.77	0.85	0.86	1.22	0.77	0.74	0.75	1.07	0.67
TER	UAA	*4.27*	*3.49*	*9.60*	*5.49*	*12.95*	*10.59*	*29.14*	*16.65*	*8.95*	*7.33*	*20.15*	*11.51*	*9.82*	*8.03*	*22.09*	*12.63*
	UAG	*2.56*	*2.56*	*3.20*	1.60	*16.54*	*16.54*	*20.68*	*10.34*	*10.22*	*10.22*	*12.78*	*6.39*	*9.82*	*9.82*	*12.27*	*6.14*
	UGA	*7.92*	*9.51*	*13.58*	*7.92*	*14.10*	*16.92*	*24.17*	*14.10*	*11.79*	*14.15*	*20.22*	*11.79*	*12.82*	*15.38*	*21.98*	*12.82*

At, Arabidopsis thaliana; Nt, Nicotiana tabacum; Pt, *Populus trichocarpa*; Os, Oryza sativa. Italic numbers in the table indicate ratios ≤ 0.5 or ≥ 2.

### Analysis of *Cis*-Acting Elements of the *JcWOXs*

**FIGURE 4:**
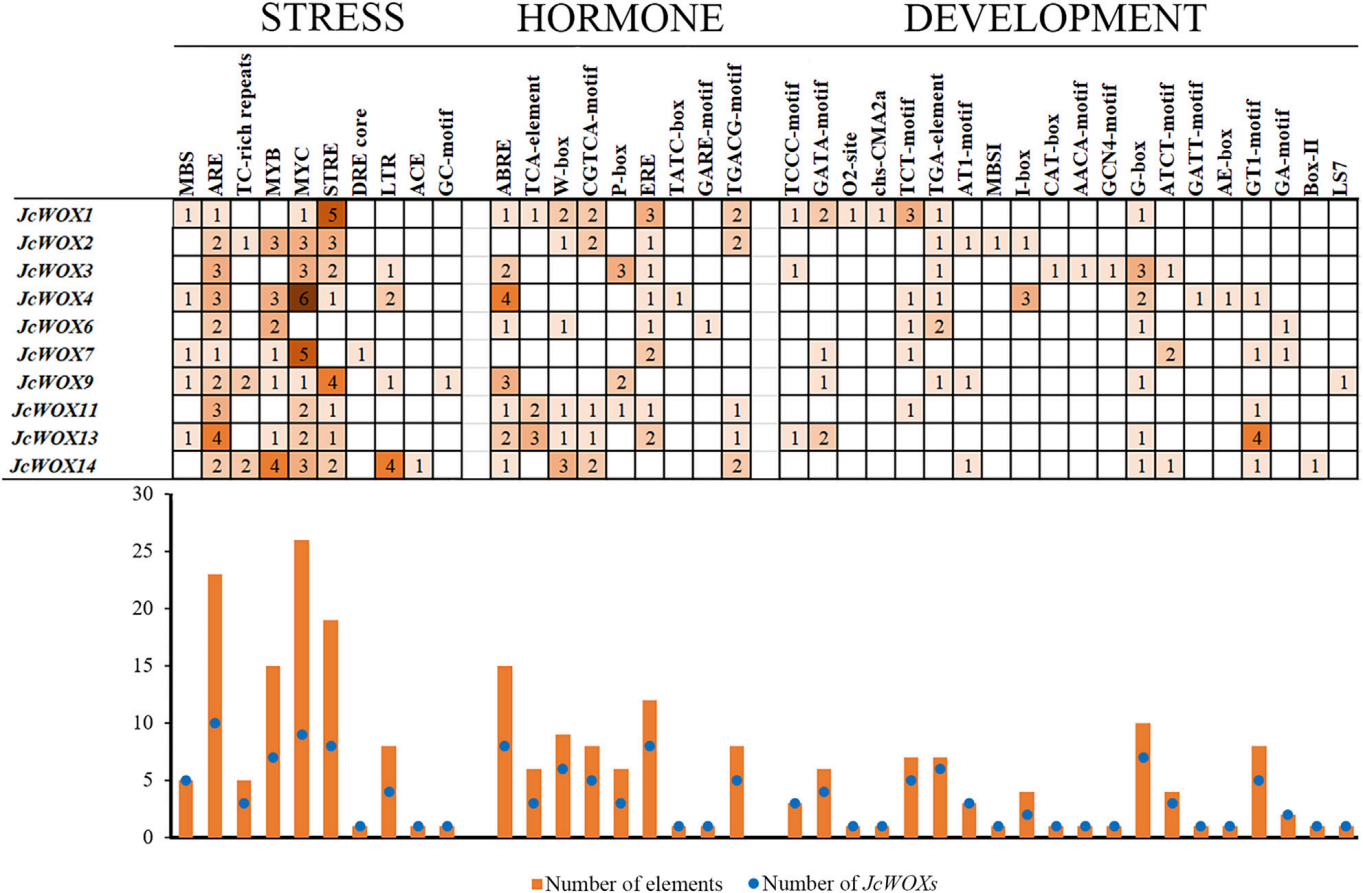
*Cis*-acting elements in the promoter regions of 10 *JcWOX* genes. The number of *cis*-acting elements related to stress was the largest.

As shown in Figure 4, the *cis*-acting elements identified in this study were divided into three types based on their functions such as stress responses, hormone responses, and growth and development. The largest group of *cis*-acting elements was related to stress at 104 among the *JcWOXs*. For most of *JcWOXs*, the number of identical *cis*-acting elements usually ranged from one to three. A large number of *cis*-acting elements were associated with growth and development—10. The most abundant *cis*-acting elements were MYCs (related to abscisic acid (ABA) induction), of which 26 existed in the promoters of nine *JcWOX* genes, excluding *JcWOX6*. Moreover, AREs (related to the anaerobic stress response) were the most widespread *cis*-acting elements in the *JcWOXs* (present within all 10 *JcWOX* gene members), suggesting that *JcWOXs* are widely involved in reactions related to anaerobic stress. In addition, *JcWOX14* contained the most stress-related *cis*-acting elements, four MYB elements (related to the drought stress response) and four LTR elements (*JcWOXs* related to the low-temperature stress responses), which reflected that *JcWOX14* plays an indispensable role in the drought stress response and low-temperature response. With respect to hormone-related promoter elements, *JcWOX1* contains the largest number six types and 11 elements—suggesting that *JcWOX1* is likely involved in hormone-related responses. ABREs (related to the ABA reaction element) were the most widespread *cis*-acting elements in the *JcWOXs* (present within eight *JcWOX* gene members, excluding *JcWOX2* and *JcWOX7*), which demonstrates that *JcWOXs* are widely involved in reactions related to ABA-related reactions. In terms of *cis*-acting elements related to growth and development, *JcWOX1* and *JcWOX4* contained the most—seven types and 10 elements. In addition, the G-box (related to the light response element) was present within seven *JcWOX* gene members, excluding *JcWOX2*, *JcWOX7*, and *JcWOX11*. Interestingly, *JcWOX13* contains four GT1 motifs that participate in the light response.

### *JcWOXs* are Specifically Involved in Spatial Expression

**FIGURE 5:**
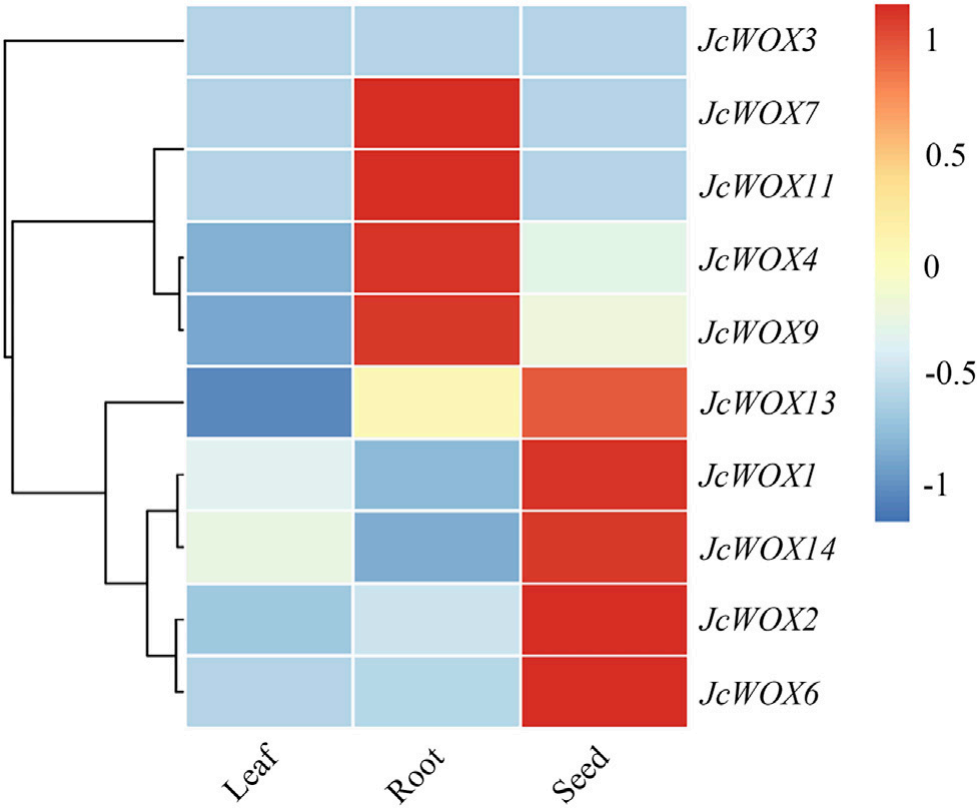
Tissue-specific expression patterns of 10 *JcWOX* genes. The role of *JcWOX* family members in the growth and development of roots and seeds may be more important than that of leaves.

We investigated previously published transcriptome profiles of various tissue types to determine the critical role of *WOX* genes in the development of *J. curcas*. Based on a heatmap of these data, we found that four genes (*JcWOX4*, *JcWOX7*, *JcWOX9*, and *JcWOX11*) in the roots and five genes (*JcWOX1*, *JcWOX2*, *JcWOX6*, *JcWOX13*, and *JcWOX14*) in the seeds. Nevertheless, *WOX* genes in *J. curcas* were expressed at low levels in the leaves (Figure 5). Based on these results, the *JcWOXs* might play more significant roles in the growth and development of roots and seeds than in leaves. In summary, the expression patterns of *JcWOXs* in different tissues respectively are conducive to identifying functional genes expressed in *J. curcas*.

### *JcWOXs* are Specifically Involved in Temporal Expression

**FIGURE 6:**
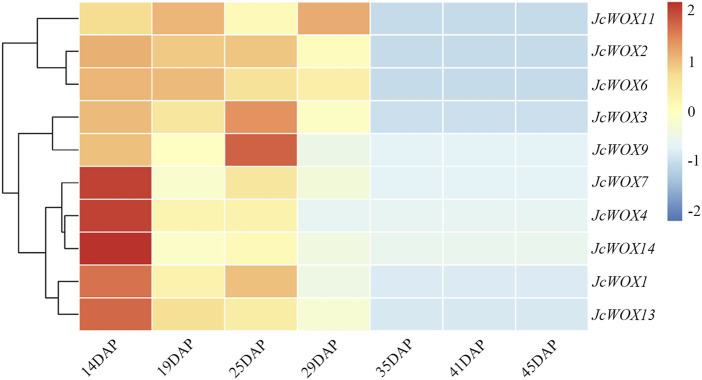
Expression patterns of 10 *JcWOX* genes during seven seed developmental stages.

Based on the analysis of the expression of *WOX* genes and their variation trends across seven *J. curcas* seed development stages (Figure 6), all *JcWOXs* were expressed at relatively high level during the initial stage (19 DAP, 25 DAP, and 29 DAP) and at relatively low level during the fast oil accumulation stage (35 DAP, 41 DAP, and 45 DAP). The expression of four *WOX* genes (*JcWOX3*, *JcWOX6*, *JcWOX11*, and *JcWOX13*) markedly decreased between 29 and 35 DAP. Interestingly, the expression of two *WOX* genes (*JcWOX6* and *JcWOX13*) decreased continuously during all seven stages. In general, the expression of *WOX* genes tended to decrease during the seven stages, hence, we deduced that *JcWOX6* and *JcWOX13* play a significant anti-regulatory role in seed development.

### qRT-PCR Analysis of *JcWOXs* Expression in Calli

**FIGURE 7:**
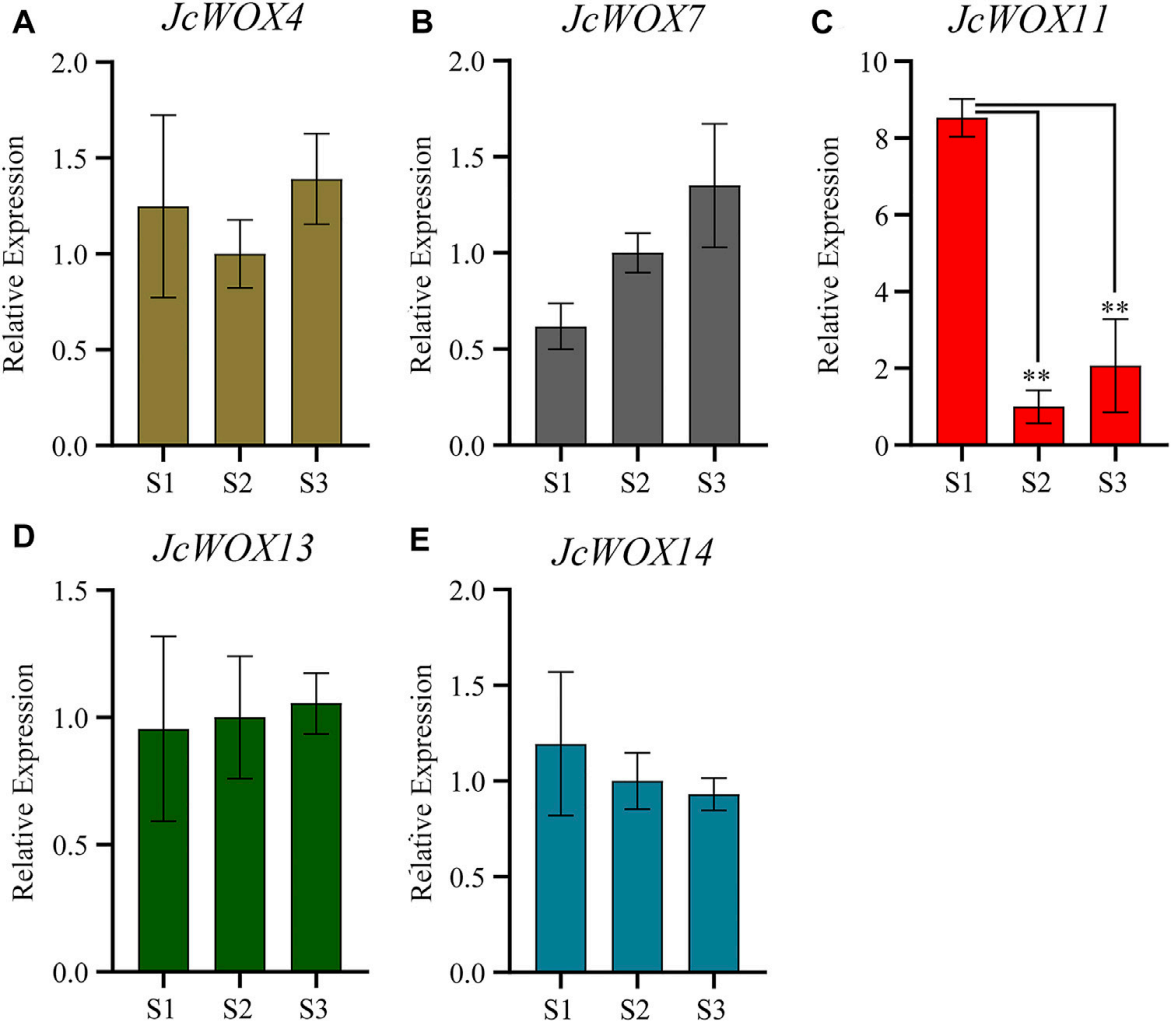
Expression of five *JcWOX* genes in callus in qRT-PCR experiments (Student’s *t*-test: ^**^ *p* < 0.01). **(A)** *JcWOX4*; **(B)** *JcWOX7*; **(C)** *JcWOX11*; **(D)** *JcWOX 13*; **(E)** *JcWOX14*.

The expression levels of *JcWOXs* were analyzed in calli collected at three stages (S1, S2, and S3) via qRT-PCR to better understand their functions. We performed qRT-PCR on *WOX* genes of *J. curcas*; the findings for some genes with polar expression levels were discarded (*JcWOX1*, *JcWOX2*, *JcWOX3*, *JcWOX6*, and *JcWOX9*). The results of qRT-PCR experiments (Figure 7) showed that among the five *WOX* genes of *J. curcas*, the expression of four *WOX* genes (*JcWOX4*, *JcWOX7*, *JcWOX13*, and *JcWOX14*) did not display obvious variation in the three biological repetitions. The expression of the *JcWOX11* gene was significantly upregulated at S1. Moreover, the gene variation in the expression of *JcWOX11* was most obvious. These findings provide new insights and a comprehensive understanding of the characteristics of *JcWOXs* for functional validation in the future.

## Discussion

In the recent study, *WOX* genes in four Euphorbiaceae species (*H. brasiliensis* (20 members), *J. curcas* (10 members), *M. esculenta* (18 members) and *R. communis* (11 members) ) were identified. Through the ExPASy analysis, we found that their protein lengths, predicted MW, and pI values did not differ substantially, and we concluded that the physicochemical properties of WOX proteins from the four Euphorbiaceae species were thermostable and hydrophilic, with very similar physicochemical properties. As plant-specific transcription factors, WOX family transcription factors are widely involved in the regulation of plant meristems and play distinct roles in the development of different tissues (Haecker et al., 2004). The function of *WOX* genes is conserved between *A. thaliana* and the four Euphorbiaceae species, and we speculated that WOX family transcription factors play a significant role in plant growth and development by regulating the subsistence of plants (Vandenbussche et al., 2009; Zhang and Tadege, 2015).

Our evolutionary tree revealed that 59 *WOX* genes were divided into three major clades. Phylogenetic analysis of *S. lycopersicum*, *O. sativa*, *A. thaliana*, and *Petunia hybrida* have also divided *WOX* genes into three separate clades: the ancient, intermediate, and modern/WUS clades (Figure 1). The ancient clade includes WOX13 and WOX14 (Lin et al., 2013; Dolzblasz et al., 2016), consistent with the evolutionary relationship between *WOX* genes in Rosaceae species (Cao et al., 2017); the intermediate clade contain WOX8, WOX9, and WOX11; and the modern/WUS clade consists of WUS and WOX1-7. Our results were consistent with the representative taxonomic results from phylogenetic analysis of *P. abies* (Hedman et al., 2013). In addition, in the ancient clade, AtWOX13 promotes fruit embryonic development (Romera-Branchat et al., 2013); in the intermediate clade, OsWOX11 is expressed specifically in the cambium and promotes adventitious root formation (Zhao et al., 2009); in the modern/WUS clade, AtWUS could maintain stem cell population stability in SAMs; and AtWOX5 has similar roles in the apical meristem (Wang et al., 2018). According to the evolutionary results of orthologous genes, JcWOX13 may be involved in fruit embryonic development, and JcWOX11 may participate in root growth.

Moreover, the genetic relationships of WOX members in different species were very close and members of the WOX families in *M. esculenta* and *H. brasiliensis* were evolutionarily close to each other in the phylogenetic tree, suggesting that these two species were more closely related than *J. curcas* and *R. communis*. These results were further supported by conserved motif analysis and gene structure analysis. The gene structure, motif number and location of each *WOX* subfamily were highly similar, which further indicated that the structure of *WOX* genes is quite conserved. The conserved motif analysis revealed that all proteins encoded by 59 *WOX* genes contained both motif 1 and motif 2, showing that these motifs are homeodomains of WOX proteins (Figure 3). We found that each clade contained specific conserved motifs, implying that these specific motifs are likely required for specific functions among the different subfamily members. For instance, motif 5 exists only in the modern/WUS clade of *WOX* genes, and the sequence represented by motif 5 is consistent with that of the WUS-box (TLXLFPXX), revealing that motif 5 is the specific WOX structural domain WUS-box, which is consistent with previous studies (Zhang et al., 2010; Cao et al., 2017; Wang et al., 2018).

Analysis of RSCU and RFSC values based on the codon bias demonstrated similarities in their codon usage of the four Euphorbiaceae species (Table 1), we mainly found that there were similarities in their codon usage, that is, there were commonalities between the four plants. We also realized that the codon usage of *WOX* genes of the Euphorbiaceae species was quite conserved. Based on the RSCU values, the four Euphorbiaceae species had 19 identical codons with positive bias. Moreover, the RFSC values reflected four identical high-frequency codons in *WOX* genes of the four Euphorbiaceae species: GCA, GAU, CCA, and AGA. After comparing the codon usage frequencies, we considered *P. trichocarpa* to be the optimal choice for comparison of exogenous *WOX* genes in *J. curcas*, *R. communis*, and *H. brasiliensis*; *N. tabacum* considered as the optimal choice for exogenous *WOX* genes in *M. esculenta*. Interestingly, *N. tabacum* to be was considered the optimal choice for comparison with the whole *J. curcas* whole genome in a previous study (Wang Z. et al., 2021), which is different from the conclusion in the present study. This result further shows the significance and value of our study of *WOX* genes and provides a direction for future research. At the same time, this finding indicates that the expression patterns receptors of exogenous genes differ for different genes even although they are from the same species.

We identified three major categories of *cis*-acting elements among *JcWOXs*: stress-related, hormone-related, and growth-related elements (Figure 4). Among them, *cis*-acting elements related to growth and development were the most abundant, with 22 types were identified. G-boxes constituted the greatest number of *cis*-acting elements associated with growth and development, which demonstrates that *WOX* genes are actively involved in light response regulation. *JcWOX14* contained the most stress-related promoters with seven types across 18 different promoters, proving that *JcWOX14* is likely involved in stress-related responses (Figure 5). This echoes a previous study of *JcWOX5* in transgenic rice in which the gene increased its sensitivity to the drought stress (Tang et al., 2020). These results confirmed the hypothesis that *JcWOXs* played a potential role in the response to abiotic stress. Additionally, *JcWOX1* contained the most hormone-related elements, with six types across 10 different promoters, suggesting that *JcWOX1* is likely involved in hormone-related responses.

Based on our expression profile, we propose that these genes may play a major part in the growth and development of roots (*JcWOX4*, *JcWOX7*, *JcWOX9*, and *JcWOX11*) or seeds (*JcWOX1*, *JcWOX2*, *JcWOX6*, *JcWOX13*, and *JcWOX14*). According to the expression analysis of *WOX* genes and their variation trends across seven *J. curcas* seed development stages, these genes play an anti-regulatory role during seed development (Figure 6). Furthermore, the expression of four *WOX* genes (*JcWOX3*, *JcWOX6*, *JcWOX11*, and *JcWOX13*) showed a significant downward trend between 29 and 35 DAP. In addition, *JcWOX6* and *JcWOX13* decreased continuously during all seven stages. Numerous studies have shown that *WOX* genes play different roles in the development of rice roots, stems, and leaves (Li et al., 2012; Wang et al., 2017; Zhou et al., 2017). The *WOX6* gene plays a significant role in the regulation of seed development, especially for the growth and development of seeds under water-deficient conditions (Shafique Khan et al., 2021). In particular, *WOX6* gene in rice regulates the asymmetric expression of auxin, resulting in the appearance of rice tiller horns (Zhang et al., 2018). *WOX13* is expressed in plant pods, flowers and seeds, with the most prominent expression in roots (Han et al., 2019). In addition, *WOX13* could be also significantly expressed in reproductive organs and the developing embryo in cotton (Yang et al., 2017). The *AtWOX13* mutant remained defective after grafting, suggesting that the *WOX13* gene is essential for tissue repair in seed plants (Ikeuchi et al., 2022). The qRT-PCR results indicated that the expression of *JcWOX11* in the callus had the most obvious change. Our results demonstrated that *JcWOX11* had the highest expression level in roots, and the expression of *JcWOX4*, *JcWOX7*, *JcWOX13*, and *JcWOX14* showed no obvious variations (Figure 7). *AtWUS* is expressed in organizing center cells of the SAM and regulates the maintenance of shoot stem cells (Dolzblasz et al., 2016). The feedback regulation mechanisms in the SAM and RAM are similar, while the expression pattern of SAM may have similarities in root apex (Wang et al., 2011). Further analysis of the shoot-containing SAM provides a new direction for our further research. In *A. thaliana*, lateral root formation can be promoted through an *AtWOX11*-mediated pathway, thus further promoting callus initiation (Kong et al., 2016; Guo et al., 2018). The high expression of *OsWOX11* enhanced the formation of adventitious roots and finally increased the uptake of nutrients by callus (Wan Abdullah et al., 2021). *OsWOX11* can integrate auxin and cytokinin signals, thereby promoting cell division during crown root development and playing a crucial role in the regulation of root development (Zhao et al., 2009). This study lays a foundation for further research in the field related to *WOX* genes and provides important reference value.

## Conclusions

In the present study, 59 *WOX* genes from four Euphorbiaceae species were identified and comprehensively analyzed to clarify their overall and molecular characteristics. Moreover, *cis*-acting elements and expression patterns of *JcWOXs* were determined under different spatiotemporal conditions. The results showed that the structures and genetic relationships of *WOX* genes in *H. brasiliensis*, *J. curcas*, *M. esculenta*, and *R. communis* providing a foundation for the functional verification of functional genes in *WOX* genes. Moreover, analyses of the spatial and temporal expression pattern analysis of *JcWOXs* in different tissues and at variety of stages of seed development indicated that two *JcWOXs* (*JcWOX*6 and *JcWOX*13) may be involved in plant growth and development. Furthermore, qRT-PCR proved that *JcWOX11* was particularly worthy of further functional analysis in promoting the callus proliferation. Overall, our study lays a foundation for future research in exploring the molecular mechanisms through which *WOX* genes drive development in Euphorbiaceae species and in other species.

## Data Availability

The original contributions presented in the study are included in the article/Supplementary Material, further inquiries can be directed to the corresponding author.

## References

[B1] Artimo P. Jonnalagedda M. Arnold K. Baratin D. Csardi G. de Castro E. (2012). ExPASy: SIB Bioinformatics Resource Portal. Nucleic Acids Res. 40, W597–W603. 10.1093/nar/gks400 22661580 PMC3394269

[B2] Bueno N. Cuesta C. Centeno M. L. Ordás R. J. Alvarez J. M. (2021). *In Vitro* Plant Regeneration in Conifers: The Role of *WOX* and *KNOX* Gene Families. Genes 12, 438. 10.3390/genes12030438 33808690 PMC8003479

[B3] Cao Y. Han Y. Meng D. Li G. Li D. Abdullah M. (2017). Genome-Wide Analysis Suggests the Relaxed Purifying Selection Affect the Evolution of *WOX* Genes in *Pyrus Bretschneideri, Prunus Persica, Prunus Mume*, and *Fragaria Vesca* . Front. Genet. 8, 78. 10.3389/fgene.2017.00078 28663757 PMC5471313

[B4] Chan A. P. Crabtree J. Zhao Q. Lorenzi H. Orvis J. Puiu D. (2010). Draft Genome Sequence of the Oilseed Species *Ricinus communis* . Nat. Biotechnol. 28, 951–956. 10.1038/nbt.1674 20729833 PMC2945230

[B5] Costanzo E. Trehin C. Vandenbussche M. (2014). The Role of *WOX* Genes in Flower Development. Ann. Bot. 114, 1545–1553. 10.1093/aob/mcu123 24973416 PMC4204783

[B6] Debnath M. Bisen P. S. (2008). *Jatropha Curcas* L., a Multipurpose Stress Resistant Plant with a Potential for Ethnomedicine and Renewable Energy. Curr. Pharm. Biotechnol. 9, 288–306. 10.2174/138920108785161541 18691089

[B7] Dolzblasz A. Nardmann J. Clerici E. Causier B. van der Graaff E. Chen J. (2016). Stem Cell Regulation by *Arabidopsis WOX* Genes. Mol. Plant 9, 1028–1039. 10.1016/j.molp.2016.04.007 27109605

[B8] Guo F. Zhang H. Liu W. Hu X. Han N. Qian Q. (2018). Callus Initiation from Root Explants Employs Different Strategies in Rice and *Arabidopsis* . Plant Cell Physiol. 59, 1782–1789. 10.1093/pcp/pcy095 29788450

[B9] Haecker A. Gross-Hardt R. Geiges B. Sarkar A. Breuninger H. Herrmann M. (2004). Expression Dynamics of *WOX* Genes Mark Cell Fate Decisions during Early Embryonic Patterning in *Arabidopsis thaliana* . Development 131, 657–668. 10.1242/dev.00963 14711878

[B10] Han N. Tang R. Chen X. Xu Z. Ren Z. Wang L. (2021). Genome-Wide Identification and Characterization of *WOX* Genes in *Cucumis Sativus* . Genome 64 (8), 761–776. 10.1139/gen-2020-0029 33493082

[B11] Han W. Li G. Feng L. Yan X. Bai Y. Li M. (2019). Genome-Wide Characterization Analysis of WOX Transcription Factors and Response to Abiotic Stresses in *Ricinus communis* L. J. Agric. Sci. 33 (10), 1921–1927. 10.11869/j.issn.100-8551.2019.10.1921

[B12] He P. Zhang Y. Liu H. Yuan Y. Wang C. Yu J. (2019). Comprehensive Analysis of *WOX* Genes Uncovers that *WOX13* is Involved in Phytohormone-Mediated Fiber Development in Cotton. BMC Plant Biol. 19, 312. 10.1186/s12870-019-1892-x 31307379 PMC6632001

[B13] Hedman H. Zhu T. von Arnold S. Sohlberg J. J. (2013). Analysis of the *WUSCHEL-RELATED HOMEOBOX* Gene Family in the Conifer *Picea Abiesreveals* Extensive Conservation as well as Dynamic Patterns. BMC Plant Biol. 13, 89. 10.1186/1471-2229-13-89 23758772 PMC3701499

[B14] Hirakawa H. Tsuchimoto S. Sakai H. Nakayama S. Fujishiro T. Kishida Y. (2012). Upgraded Genomic Information of *Jatropha Curcas* L. Plant Biotechnol. 29, 123–130. 10.5511/plantbiotechnology.12.0515a

[B15] Hu B. Jin J. Guo A. Zhang H. Luo J. Gao G. (2015). GSDS 2.0: An Upgraded Gene Feature Visualization Server. Bioinformatics 31, 1296–1297. 10.1093/bioinformatics/btu817 25504850 PMC4393523

[B16] Ikeuchi M. Iwase A. Ito T. Tanaka H. Favero D. S. Kawamura A. (2022). Wound-Inducible WUSCHEL-RELATED HOMEOBOX 13 is Required for Callus Growth and Organ Reconnection. Plant Physiol. 188, 425–441. 10.1093/plphys/kiab510 34730809 PMC8774835

[B17] Jiang H. Wu P. Zhang S. Song C. Chen Y. Li M. (2012). Global Analysis of Gene Expression Profiles in Developing Physic Nut (*Jatropha Curcas* L.) Seeds. PLoS One 7, e36522. 10.1371/journal.pone.0036522 22574177 PMC3344900

[B18] Kong D. Hao Y. Cui H. (2016). The WUSCHEL Related Homeobox Protein WOX7 Regulates the Sugar Response of Lateral Root Development in *Arabidopsis thaliana* . Mol. Plant 9, 261–270. 10.1016/j.molp.2015.11.006 26621542

[B19] Kumar S. Stecher G. Li M. Knyaz C. Tamura K. (2018). MEGA X: Molecular Evolutionary Genetics Analysis across Computing Platforms. Mol. Biol. Evol. 35, 1547–1549. 10.1093/molbev/msy096 29722887 PMC5967553

[B20] Lescot M. Déhais P. Thijs G. Marchal K. Moreau Y. Van de Peer Y. (2002). PlantCARE, a Database of Plant *Cis*-Acting Regulatory Elements and a Portal to Tools for *in Silico* Analysis of Promoter Sequences. Nucleic Acids Res. 30, 325–327. 10.1093/nar/30.1.325 11752327 PMC99092

[B21] Li J. Gao X. Sang S. Liu C. (2019). Genome-Wide Identification, Phylogeny, and Expression Analysis of the SBP-Box Gene Family in Euphorbiaceae. BMC Genomics 20, 912. 10.1186/s12864-019-6319-4 31874634 PMC6929338

[B22] Li J. Yuan Y. Lu Z. Yang L. Gao R. Lu J. (2012). *Glabrous Rice* 1, Encoding a Homeodomain Protein, Regulates Trichome Development in Rice. Rice 5, 1–10. 10.1186/1939-8433-5-32 24279910 PMC4883694

[B23] Li X. Hamyat M. Liu C. Ahmad S. Gao X. Guo C. (2018). Identification and Characterization of the WOX Family Genes in Five *Solanaceae* Species Reveal Their Conserved Roles in Peptide Signaling. Genes 9, 260. 10.3390/genes9050260 PMC5977200 29772825

[B24] Li X. Liu C. Li W. Zhang Z. Gao X. Zhou H. (2016). Genome-Wide Identification, Phylogenetic Analysis and Expression Profiling of the WOX Family Genes in *Solanum lycopersicum* . Hereditas. 38, 444–460. 10.16288/j.yczz.15-499 27232493

[B25] Lin H. Niu L. McHale N. A. Ohme-Takagi M. Mysore K. S. Tadege M. (2013). Evolutionarily Conserved Repressive Activity of WOX Proteins Mediates Leaf Blade Outgrowth and Floral Organ Development in Plants. Proc. Natl. Acad. Sci. U.S.A. 110, 366–371. 10.1073/pnas.1215376110 23248305 PMC3538250

[B26] Liu B. Wang W. Gao J. Chen F. Wang S. Xu Y. (2010). Molecular Cloning and Characterization of a Jasmonate Biosynthetic Pathway Gene for Allene Oxide Cyclase from *Jatropha Curcas* . Acta Physiol. Plant. 32, 531–539. 10.1007/s11738-009-0430-0

[B27] Liu J. Sheng L. Xu Y. Li J. Yang Z. Huang H. (2014). *WOX11* and *12* are Involved in the First-Step Cell Fate Transition during de Novo Root Organogenesis in *Arabidopsis* . Plant Cell 26 (3), 1081–1093. 10.1105/tpc.114.122887 24642937 PMC4001370

[B28] Maghuly F. Laimer M. (2013). *Jatropha Curcas*, a Biofuel Crop: Functional Genomics for Understanding Metabolic Pathways and Genetic Improvement. Biotechnol. J. 8, 1172–1182. 10.1002/biot.201300231 24092674 PMC4065342

[B29] Natarajan P. Parani M. (2011). *De Novo* Assembly and Transcriptome Analysis of Five Major Tissues of *Jatropha Curcas* L. Using GS FLX Titanium Platform of 454 Pyrosequencing. BMC Genomics 12, 191. 10.1186/1471-2164-12-191 21492485 PMC3087711

[B30] Palovaara J. Hallberg H. Stasolla C. Hakman I. (2010). Comparative Expression Pattern Analysis of *WUSCHEL-Related Homeobox 2* (*WOX2*) and *WOX8/9* in Developing Seeds and Somatic Embryos of the Gymnosperm *Picea Abies* . New Phytol. 188, 122–135. 10.1111/j.1469-8137.2010.03336.x 20561212

[B31] Prochnik S. Marri P. R. Desany B. Rabinowicz P. D. Kodira C. Mohiuddin M. (2012). The Cassava Genome: Current Progress, Future Directions. Trop. Plant Biol. 5, 88–94. 10.1007/s12042-011-9088-z 22523606 PMC3322327

[B32] Rahman A. Y. A. Usharraj A. O. Misra B. B. Thottathil G. P. Jayasekaran K. Feng Y. (2013). Draft Genome Sequence of the Rubber Tree *Hevea Brasiliensis* . BMC Genomics. 14, 75. 10.1186/1471-2164-14-75 23375136 PMC3575267

[B33] Rehn L. S. Rodrigues A. A. Vasconcelos-Filho S. C. Rodrigues D. A. de Freitas Moura L. M. Costa A. C. (2020). *Ricinus communis* as a Phytoremediator of Soil Mineral Oil: Morphoanatomical and Physiological Traits. Ecotoxicology 29, 129–139. 10.1007/s10646-019-02147-6 31865512

[B34] Romera-Branchat M. Ripoll J. J. Yanofsky M. F. Pelaz S. (2013). The *WOX13* Homeobox Gene Promotes Replum Formation in the *Arabidopsis Thaliana* Fruit. Plant J. 73, 37–49. 10.1111/tpj.12010 22946675

[B35] Santos-Silva C. A. D. Vilela L. M. B. Oliveira-Silva R. L. d. Silva J. B. D. Machado A. R. Bezerra-Neto J. P. (2021). Cassava (*Manihot Esculenta*) Defensins: Prospection, Structural Analysis and Tissue-Specific Expression under Biotic/Abiotic Stresses. Biochimie 186, 1–12. 10.1016/j.biochi.2021.03.012 33789147

[B36] Shafique Khan F. Zeng R. Gan Z. Zhang J. Hu C. (2021). Genome-Wide Identification and Expression Profiling of the *WOX* Gene Family in *Citrus Sinensis* and Functional Analysis of a *CsWUS* Member. Int. J. Mol. Sci. 22, 4919. 10.3390/ijms22094919 34066408 PMC8124563

[B37] Shi L. Wang K. Du L. Song Y. Li H. Ye X. (2021). Genome-Wide Identification and Expression Profiling Analysis of WOX Family Protein-Encoded Genes in Triticeae Species. Int. J. Mol. Sci. 22, 9325. 10.3390/ijms22179325 34502234 PMC8431079

[B38] Supriya R. Priyadarshan P. M. (2019). Genomic Technologies for *Hevea* Breeding. Adv. Genet. 104, 1–73. 10.1016/bs.adgen.2019.04.001 31200808

[B39] Tang Y. Bao X. Jian W. Lou H. Feng Y. Jie T. (2019). Genome-Wide Identification and Expression Analysis of the *WOX* Gene Family in Physic Nut. Mol. Plant Breed. 17, 1154–1162. 10.13271/j.mpb.017.001154

[B40] Tang Y. Li H. Guan Y. Li S. Xun C. Dong Y. (2020). Genome-Wide Identification of the Physic Nut WUSCHEL-Related Homeobox Gene Family and Functional Analysis of the Abiotic Stress Responsive Gene *JcWOX5* . Front. Genet. 11, 670. 10.3389/fgene.2020.00670 32655627 PMC7325900

[B41] Thompson J. D. Gibson T. J. Plewniak F. Jeanmougin F. Higgins D. G. (1997). The CLUSTAL_X Windows Interface: Flexible Strategies for Multiple Sequence Alignment Aided by Quality Analysis Tools. Nucleic acids. Res. 25, 4876–4882. 10.1093/nar/25.24.4876 9396791 PMC147148

[B42] Tvorogova V. E. Krasnoperova E. Y. Potsenkovskaia E. A. Kudriashov A. A. Dodueva I. E. Lutova L. A. (2021). What Does the WOX Say? Review of Regulators, Targets, Partners. Mol. Biol. Mosk. 55, 362–391. 10.31857/S0026898421030174 34097673

[B43] Ueda M. Zhang Z. Laux T. (2011). Transcriptional Activation of *Arabidopsis* Axis Patterning Genes *WOX8/9* Links Zygote Polarity to Embryo Development. Dev. Cell 20, 264–270. 10.1016/j.devcel.2011.01.009 21316593

[B44] Vandenbussche M. Horstman A. Zethof J. Koes R. Rijpkema A. S. Gerats T. (2009). Differential Recruitment of *WOX* Transcription Factors for Lateral Development and Organ Fusion in Petunia and *Arabidopsis* . Plant Cell 21 (8), 2269–2283. 10.1105/tpc.109.065862 19717616 PMC2751957

[B45] Wan Abdullah W. M. A. N. Tan N. P. Low L. Y. Loh J. Y. Wee C. Y. Md Taib A. Z. (2021). Calcium Lignosulfonate Improves Proliferation of Recalcitrant Indica Rice Callus via Modulation of Auxin Biosynthesis and Enhancement of Nutrient Absorption. Plant Physiology Biochem. 161, 131–142. 10.1016/j.plaphy.2021.01.046 33581621

[B46] Wang D. Hao Z. Long X. Wang Z. Zheng X. Ye D. (2020). The Transcriptome of *Cunninghamia Lanceolata* Male/Female Cone Reveal the Association between MIKC MADS-Box Genes and Reproductive Organs Development. BMC Plant Biol. 20, 508. 10.1186/s12870-020-02634-7 33153428 PMC7643283

[B47] Wang H. Niu H. Li C. Shen G. Liu X. Weng Y. (2020). WUSCHEL-Related Homeobox1 (WOX1) Regulates Vein Patterning and Leaf Size in *Cucumis Sativus* . Hortic. Res. 7, 182. 10.1038/s41438-020-00404-y 33328463 PMC7603520

[B48] Wang X. Bi C. Wang C. Ye Q. Yin T. Ye N. (2018). Genome-Wide Identification and Characterization of *WUSCHEL-*Related Homeobox (*WOX*) Genes in *Salix Suchowensis* . J. For. Res. 30, 1811–1822. 10.1007/s11676-018-0734-2

[B49] Wang Y. Li H. Zhou Y. Guo D. Zhu J. Peng S. (2021). Transcriptomes Analysis Reveals Novel Insight into the Molecular Mechanisms of Somatic Embryogenesis in *Hevea Brasiliensis* . BMC Genomics 22, 183. 10.1186/s12864-021-07501-9 33711923 PMC7953812

[B50] Wang Y. Song F. Zhu J. Zhang S. Yang Y. Chen T. (2017). GSA: Genome Sequence Archive. Genom. Proteom. Bionf. 15, 14–18. 10.1016/j.gpb.2017.01.001 PMC5339404 28387199

[B51] Wang Z. Chen J. Shi J. (2011). Progress on *WUS/CLV* Feedback Regulatory Mechanisms in Plant Stem Cells. Sci. Silvae Sin. 47 (004), 159–165. 10.3724/SP.J.1011.2011.00353

[B52] Wang Z. Wang G. Cai Q. Jiang Y. Wang C. Xia H. (2021). Genomewide Comparative Analysis of Codon Usage Bias in Three Sequenced *Jatropha Curcas* . J. Genet. 100, 20. 10.1007/s12041-021-01271-9 34057149

[B53] Webster G. L. (1994). Classification of the Euphorbiaceae. Ann. Mo. Botanical Gard. 81, 3–32. 10.2307/2399908

[B54] Wu P. Zhou C. Cheng S. Wu Z. Lu W. Han J. (2015). Integrated Genome Sequence and Linkage Map of Physic Nut (*Jatropha Curcas* L.), a Biodiesel Plant. Plant J. 81, 810–821. 10.1111/tpj.12761 25603894

[B55] Wu X. Chory J. Weigel D. (2007). Combinations of WOX Activities Regulate Tissue Proliferation during *Arabidopsis* Embryonic Development. Dev. Biol. 309, 306–316. 10.1016/j.ydbio.2007.07.019 17706632 PMC2692342

[B56] Yang R. Wu Z. Bai C. Sun Z. Wang M. Huo Y. (2021). Overexpression of *PvWOX3a* in Switchgrass Promotes Stem Development and Increases Plant Height. Hortic. Res. 8, 252. 10.1038/s41438-021-00678-w 34848686 PMC8633294

[B57] Yang Z. Gong Q. Qin W. Yang Z. Cheng Y. Lu L. (2017). Genome-Wide Analysis of *WOX* Genes in Upland Cotton and Their Expression Pattern under Different Stresses. BMC Plant Biol. 17, 113. 10.1186/s12870-017-1065-8 28683794 PMC5501002

[B58] Yoo S. C. Cho S. H. Paek N. C. (2013). Rice WUSCHEL-Related Homeobox 3A (OsWOX3A) Modulates Auxin-Transport Gene Expression in Lateral Root and Root Hair Development. Plant Signal. Behav. 8, e25929. 10.4161/psb.25929 PMC4091085 24002214

[B59] Zhang F. Tadege M. (2015). Repression of *AS2* by WOX Family Transcription Factors is Required for Leaf Development in Medicago and Arabidopsis. Plant Signal. Behav. 10, e993291. 10.4161/15592324.2014.993291 25807065 PMC4623463

[B60] Zhang L. He L. Fu Q. Xu Z. (2013). Selection of Reliable Reference Genes for Gene Expression Studies in the Biofuel Plant *Jatropha Curcas* Using Real-Time Quantitative PCR. Int. J. Mol. Sci. 14, 24338–24354. 10.3390/ijms141224338 24351820 PMC3876114

[B61] Zhang N. Yu H. Yu H. Cai Y. Huang L. Xu C. (2018). A Core Regulatory Pathway Controlling Rice Tiller Angle Mediated by the *LAZY1*-Dependent Asymmetric Distribution of Auxin. Plant Cell 30, 1461–1475. 10.1105/tpc.18.00063 29915152 PMC6096585

[B62] Zhang X. Zong J. Liu J. Yin J. Zhang D. (2010). Genome-Wide Analysis of *WOX* Gene Family in Rice, Sorghum, Maize, *Arabidopsis* and Poplar. J. Integr. Plant Biol. 52, 1016–1026. 10.1111/j.1744-7909.2010.00982.x 20977659

[B63] Zhao S. Jiang Q. Ma J. Zhang X. Zhao Q. Wang X. (2014). Characterization and Expression Analysis of *WOX5* Genes from Wheat and its Relatives. Gene 537, 63–69. 10.1016/j.gene.2013.12.022 24368329

[B64] Zhao Y. Hu Y. Dai M. Huang L. Zhou D. (2009). The WUSCHEL-Related Homeobox Gene *WOX11* is Required to Activate Shoot-Borne Crown Root Development in Rice. Plant Cell 21, 736–748. 10.1105/tpc.108.061655 19258439 PMC2671696

[B66] Zhou M. Tong C. Shi J. (2007a). Analysis of Codon Usage between Different Poplar Species. J. Genet. Genomics 34, 555–561. 10.1016/S1673-8527(07)60061-7 17601615

[B65] Zhou M. Tong C. Shi J. (2007b). A Preliminary Analysis of Synonymous Codon Usage in Poplar Species. Zhi Wu Sheng Li Yu Fen Zi Sheng Wu Xue Xue Bao 33 (4), 285–293. 10.2471/BLT.13.118778 17675751

[B67] Zhou S. Jiang W. Long F. Cheng S. Yang W. Zhao Y. (2017). Rice Homeodomain Protein WOX11 Recruits a Histone Acetyltransferase Complex to Establish Programs of Cell Proliferation of Crown Root Meristem. Plant Cell 29, 1088–1104. 10.1105/tpc.16.00908 28487409 PMC5466029

[B68] Zou Z. Yang L. Gong J. Mo Y. Wang J. Cao J. (2016). Genome-Wide Identification of *Jatropha Curcas* Aquaporin Genes and the Comparative Analysis Provides Insights into the Gene Family Expansion and Evolution in *Hevea Brasiliensis* . Front. Plant Sci. 7, 395. 10.3389/fpls.2016.00395 27066041 PMC4814485

