# Supplementary material for: Genome-Wide Identification and Comparative Analysis of WOX Genes in Four Euphorbiaceae Species and Their Expression Patterns in Jatropha curcas
Source: Front Genet. 2022 Jun 30;13:878554. doi: 10.3389/fgene.2022.878554 (PMC9280045; doi:10.3389/fgene.2022.878554)
Supplement: Supplementary file 1 [file Table1.DOCX]

**Table S1.** The transcription accession of *JcWOX* genes expression analysis.

| Number | Transcriptional sample description | Sample | |
| --- | --- | --- | --- |
|  |  | Name | Accession |
|  | Three tissues | Leaf | SRX750580 |
| a |  | Root | SRX750579 |
|  |  | Seed | SRX750581 |
|  |  | 14DAP | SRX809778 |
|  |  | 19DAP | SRX809781 |
|  |  | 25DAP | SRX809783 |
| b | The seeds at different developmental stages | 29DAP | SRX809785 |
|  |  | 35DAP | SRX809786 |
|  |  | 41DAP | SRX809787 |
|  |  | 45DAP | SRX809788 |

DAP：days after pollination.
