# Supplementary figures and images for: Genome-Wide Identification and Comparative Analysis of WOX Genes in Four Euphorbiaceae Species and Their Expression Patterns in Jatropha curcas
Source: Front Genet. 2022 Jun 30;13:878554. doi: 10.3389/fgene.2022.878554 (PMC9280045; doi:10.3389/fgene.2022.878554)

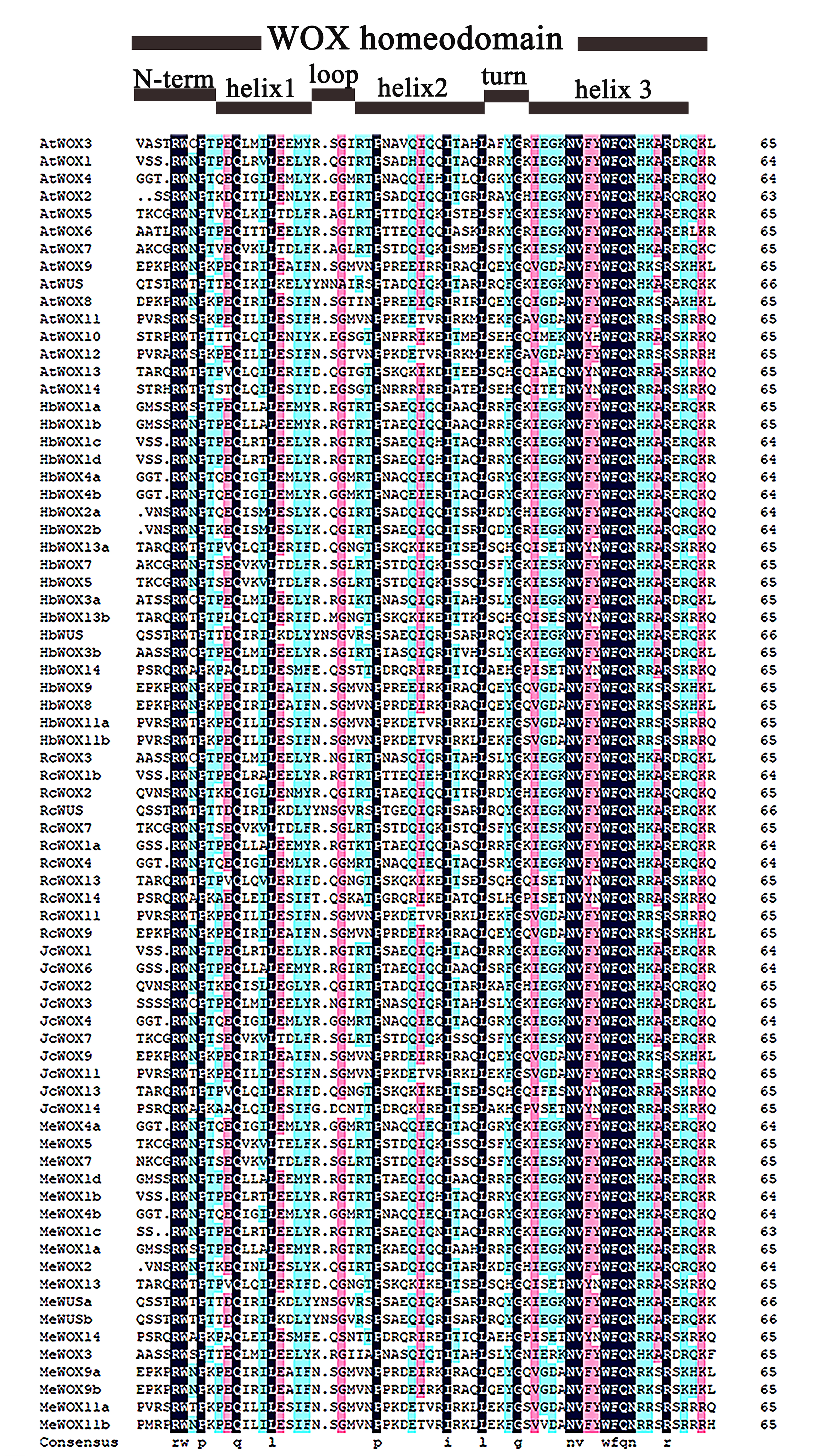

Supplement: Supplementary file 2 [file Image2.JPEG]

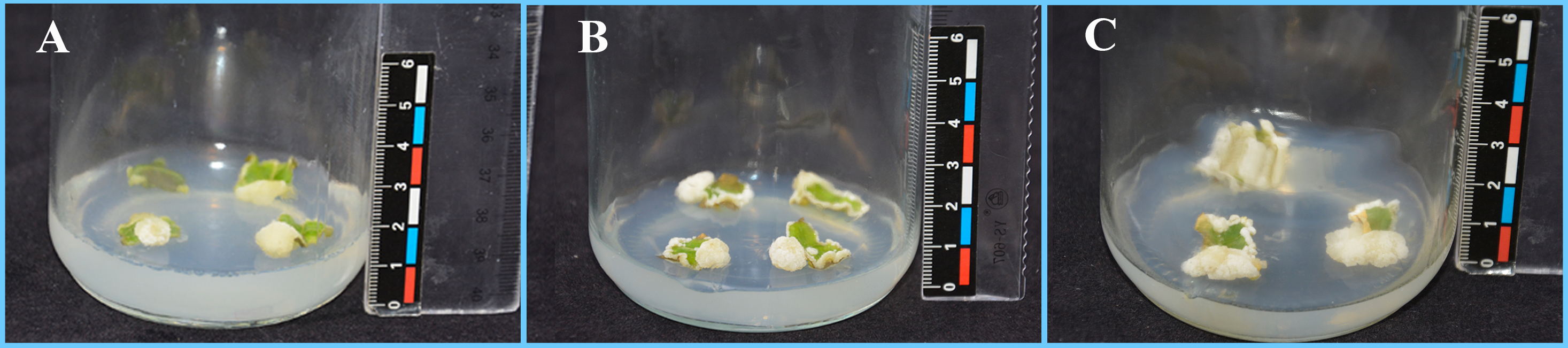

Supplement: Supplementary file 3 [file Image1.TIF]
