# Supplementary material for: Genome-Wide Identification and Comparative Analysis of WOX Genes in Four Euphorbiaceae Species and Their Expression Patterns in Jatropha curcas
Source: Front Genet. 2022 Jun 30;13:878554. doi: 10.3389/fgene.2022.878554 (PMC9280045; doi:10.3389/fgene.2022.878554)
Supplement: Supplementary file 4 [file Table2.DOCX]

**Table S2.** Details of primers for qRT-PCR analysis of *JcWOX* genes in callus.

| **Gene Name** | **Gene ID** | **Primer sequences** |
| --- | --- | --- |
| *JcGAPDH* | Jcr4U29393.10 | F: 5'-TGAAGGACTGGAGAGGTGGAAGAGC-3'  R: 5'-ATCAACAGTTGGAACACGGAAAGCC-3' |
| *JcActin* | Jcr4S06558.10 | F: 5'-CTCCTCTCAACCCCAAAGCCAA-3'  R: 5'-CACCAGAATCCAGCACGATACCA-3' |
| *JcWOX4* | Jcr4S01288.30 | F: 5'-ATGAGAACTCCTAATGCCCAA-3'  R: 5'-TGCACTTTCTCTTGTATGGAC-3' |
| *JcWOX7* | Jcr4S05032.10 | F: 5'-CTGGACTCCGAACCCCAAGCA-3'  R: 5'-CGACGTTTCTGCCTTTCTCTAGC-3' |
| *JcWOX11* | Jcr4S03419.60 | F: 5'-CGACGGTCAAGATCTCGCCG-3'  R: 5'-CTGCAGCTGCCACCACCTAG-3' |
| *JcWOX13* | Jcr4S03185.10 | F: 5'-GATTGCTGTTTATGCCACT-3'  R: 5'-ATTAATGGGTCACAGTACAGA-3' |
| *JcWOX14* | Jcr4S00240.250 | F: 5'-GGTTTGCAGAGAATCCCTTCGAG-3'  R: 5'-GACCATGTTTTGCAAGTTCACTCG-3' |
