# Supplementary material for: Genome-Wide Identification and Comparative Analysis of WOX Genes in Four Euphorbiaceae Species and Their Expression Patterns in Jatropha curcas
Source: Front Genet. 2022 Jun 30;13:878554. doi: 10.3389/fgene.2022.878554 (PMC9280045; doi:10.3389/fgene.2022.878554)
Supplement: Supplementary file 5 [file Table3.DOCX]

**Table S3.** Detailed characteristics of WOX proteins identified in four Euphorbiaceae species.

| **Gene ID** | **Chromosomal location** | **Protein**  **length/aa** | **Base pairs/bp** | **Molecular**  **weight**  **(Mw)/Da** | **Isoelectric point**  **(pI)** | **Instability Index (II)** | **Grand average of**  **hydropathicity (GRAVY)** | **Aliphaticindex (AI)** |
| --- | --- | --- | --- | --- | --- | --- | --- | --- |
| HbWUS | LOC110658826:645886-647721(+) | 285 | 858 | 32029.93 | 5.87 | 48.30 | -1.013 | 44.49 |
| HbWOX1a | LOC110663193:303915-306155(+) | 344 | 1035 | 39297.98 | 8.94 | 66.74 | -0.857 | 59.83 |
| HbWOX1b | LOC110651003:21628-23940(+) | 351 | 1056 | 40220.89 | 8.10 | 66.91 | -0.916 | 57.75 |
| HbWOX1c | LOC110647789:58292-60943(+) | 387 | 1164 | 44058.10 | 8.78 | 61.44 | -0.877 | 58.04 |
| HbWOX1d | LOC110637753:1655-4422(-) | 382 | 1149 | 43240.09 | 6.68 | 57.25 | -0.793 | 58.04 |
| HbWOX2a | LOC110666118:1225324-1226579(+) | 242 | 729 | 27205.38 | 8.95 | 59.84 | -0.761 | 60.08 |
| HbWOX2b | LOC110632678:562172-563545(+) | 237 | 714 | 26668.97 | 8.95 | 64.99 | -0.722 | 61.31 |
| HbWOX3a | LOC110646102:148080-148776(+) | 197 | 594 | 23019.30 | 9.00 | 73.62 | -0.707 | 74.31 |
| HbWOX3b | LOC110654448:2171319-2172076(-) | 217 | 654 | 25170.71 | 9.03 | 70.25 | -0.744 | 63.82 |
| HbWOX4a | LOC110671166:486102-487442(-) | 212 | 639 | 24331.44 | 9.21 | 54.00 | -0.973 | 62.12 |
| HbWOX4b | LOC110651283:7020-8255(-) | 209 | 630 | 23932.06 | 9.51 | 59.85 | -1.010 | 61.63 |
| HbWOX5 | LOC110640963:286732-287639(-) | 185 | 558 | 21558.16 | 8.35 | 64.95 | -0.831 | 58.43 |
| HbWOX7 | LOC110653501:2643325-2644109(-) | 187 | 564 | 21346.99 | 8.26 | 68.03 | -0.723 | 60.43 |
| HbWOX8 | LOC110633278:415955-418429(-) | 398 | 1197 | 43828.85 | 8.18 | 58.05 | -0.524 | 64.85 |
| HbWOX9 | LOC110638249:345666-348750(-) | 383 | 1152 | 42002.70 | 6.97 | 53.48 | -0.523 | 65.12 |
| HbWOX11a | LOC110655693:1933011-1935427(+) | 254 | 765 | 27745.00 | 6.38 | 70.42 | -0.428 | 66.34 |
| HbWOX11b | LOC110641254:222334-223734(-) | 259 | 780 | 28082.31 | 5.63 | 62.76 | -0.388 | 69.54 |
| HbWOX13a | LOC110660795:2235493-2238506(+) | 256 | 771 | 29024.05 | 5.64 | 51.29 | -0.991 | 63.98 |
| HbWOX13b | LOC110672819:113073-116219(+) | 253 | 762 | 29039.31 | 5.64 | 52.49 | -0.913 | 68.97 |
| HbWOX14 | LOC110669207:364122-365541(+) | 214 | 645 | 24446.47 | 5.64 | 55.60 | -0.735 | 69.30 |
| JcWOX1 | Jcr4S08740.10:4078-6291(-) | 392 | 1179 | 43900.77 | 7.65 | 61.18 | -0.824 | 56.81 |
| JcWOX2 | Jcr4S00826.20:19545-20735(-) | 212 | 639 | 24018.24 | 9.35 | 61.10 | -0.683 | 62.12 |
| JcWOX3 | Jcr4S01144.40:18845-20279(+) | 246 | 741 | 28357.10 | 7.89 | 60.05 | -0.663 | 65.49 |
| JcWOX4 | Jcr4S01288.30:20312-21155(+) | 217 | 654 | 24889.00 | 9.51 | 53.13 | -1.057 | 57.97 |
| JcWOX6 | Jcr4S08457.10:1298-3070(-) | 286 | 861 | 33669.17 | 9.06 | 61.03 | -0.941 | 61.75 |
| JcWOX7 | Jcr4S05032.10:508-1230(-) | 190 | 573 | 21747.21 | 9.19 | 62.43 | -0.923 | 50.21 |
| JcWOX9 | Jcr4S03146.130:45224-47129(-) | 380 | 1143 | 41708.48 | 8.24 | 61.53 | -0.525 | 64.84 |
| JcWOX11 | Jcr4S03419.60:18841-20269(+) | 267 | 804 | 28980.33 | 5.69 | 69.03 | -0.365 | 70.37 |
| JcWOX13 | Jcr4S03185.10:1987-4260(+) | 258 | 777 | 29281.41 | 5.35 | 51.92 | -0.957 | 63.84 |
| JcWOX14 | Jcr4S00240.250:105068-105871(-) | 208 | 627 | 23715.68 | 5.15 | 42.28 | -0.645 | 72.21 |
| MeWUSa | LOC110608532:7788156-7789460(+) | 288 | 867 | 32625.69 | 6.24 | 57.60 | -0.971 | 52.47 |
| MeWUSb | LOC110611794:25717905-25719663(+) | 337 | 1014 | 37984.10 | 5.61 | 53.18 | -0.637 | 57.63 |
| MeWOX1a | LOC110602182:9839801-9841540(-) | 301 | 906 | 34638.18 | 8.59 | 71.83 | -0.801 | 66.11 |
| MeWOX1b | LOC110600389:23781424-23784243(-) | 391 | 1176 | 44320.23 | 6.77 | 59.75 | -0.846 | 57.70 |
| MeWOX1c | LOC110617095:1181049-1183912(+) | 387 | 1164 | 44135.09 | 6.36 | 59.02 | -0.841 | 58.29 |
| MeWOX1d | LOC110604916:21694583-21696336(-) | 295 | 888 | 34247.32 | 7.68 | 60.90 | -0.967 | 57.19 |
| MeWOX2 | LOC110614026:3829854-3831270(-) | 246 | 741 | 27741.88 | 6.90 | 57.51 | -0.741 | 60.69 |
| MeWOX3 | LOC110614126:3015004-3015957(-) | 182 | 549 | 20744.81 | 9.07 | 70.79 | -0.497 | 82.58 |
| MeWOX4a | LOC110606337:3482903-3484137(-) | 212 | 639 | 24412.53 | 9.40 | 59.54 | -0.949 | 63.96 |
| MeWOX4b | LOC110614815:24242060-24243742(-) | 212 | 639 | 24375.49 | 9.21 | 61.88 | -1.035 | 60.28 |
| MeWOX5 | LOC110623981:1322585-1323548(+) | 231 | 696 | 26568.96 | 8.54 | 57.51 | -0.623 | 71.34 |
| MeWOX7 | LOC110618433:25193051-25193894(-) | 182 | 549 | 21317.79 | 6.76 | 67.04 | -0.89 | 57.80 |
| MeWOX9a | LOC110603549:3763479-3766645(-) | 388 | 1167 | 42527.38 | 6.78 | 51.93 | -0.495 | 63.53 |
| MeWOX9b | LOC110605037:18868484-18871545(-) | 386 | 1161 | 42436.59 | 8.15 | 51.05 | -0.438 | 67.67 |
| MeWOX11a | LOC110621677:31641397-31643436(-) | 254 | 756 | 27731.92 | 6.17 | 67.18 | -0.422 | 63.66 |
| MeWOX11b | LOC110623787:25632348-25634720(-) | 260 | 783 | 28241.46 | 5.82 | 63.10 | -0.428 | 70.85 |
| MeWOX13 | LOC110609985:8493497-8496640(+) | 251 | 756 | 29154.26 | 5.65 | 47.84 | -0.962 | 65.64 |
| MeWOX14 | LOC110614870:14891526-14892896(+) | 276 | 831 | 31666.05 | 6.51 | 53.13 | -0.522 | 75.22 |
| RcWUS | LOC8267096:80660-82938(-) | 297 | 894 | 33708.44 | 5.84 | 62.56 | -1.163 | 44.34 |
| RcWOX1a | LOC8258203:318726-320494(-) | 316 | 951 | 36360.74 | 9.42 | 59.87 | -0.962 | 57.72 |
| RcWOX1b | LOC8286328:7065-9869(+) | 401 | 1206 | 45168.80 | 6.71 | 53.52 | -0.874 | 51.67 |
| RcWOX2 | LOC8269403:21456-22774(+) | 268 | 807 | 30499.86 | 7.63 | 57.41 | -0.893 | 60.78 |
| RcWOX3 | LOC8278044:88353-89568(-) | 223 | 672 | 26170.76 | 7.33 | 60.92 | -0.823 | 59.55 |
| RcWOX4 | LOC8288579:1301360-1302605(+) | 228 | 959 | 25956.24 | 9.17 | 57.08 | -0.951 | 60.75 |
| RcWOX7 | LOC8270437:81350-82309(-) | 192 | 579 | 22050.46 | 8.60 | 47.41 | -0.933 | 54.27 |
| RcWOX9 | LOC8259173:92359-94804(-) | 390 | 1173 | 42991.91 | 8.10 | 60.88 | -0.588 | 58.74 |
| RcWOX11 | LOC8282397:6310-9891(-) | 260 | 786 | 28452.71 | 5.83 | 65.07 | -0.429 | 65.29 |
| RcWOX13 | LOC8281315:528157-530917(+) | 244 | 753 | 28246.42 | 6.25 | 52.31 | -0.758 | 74.84 |
| RcWOX14 | LOC8273628:4246511-4247946(+) | 231 | 696 | 26493.89 | 5.35 | 60.14 | -0.743 | 70.52 |
